# Overexpression of the Endoplasmic Reticulum Chaperone BiP3 Regulates XA21-Mediated Innate Immunity in Rice

**DOI:** 10.1371/journal.pone.0009262

**Published:** 2010-02-17

**Authors:** Chang-Jin Park, Rebecca Bart, Mawsheng Chern, Patrick E. Canlas, Wei Bai, Pamela C. Ronald

**Affiliations:** Department of Plant Pathology, University of California Davis, Davis, California, United States of America; Cairo University, Egypt

## Abstract

Recognition of pathogen-associated molecular patterns by pattern recognition receptors (PRRs) activates the innate immune response. Although PRR-mediated signaling events are critical to the survival of plants and animals, secretion and localization of PRRs have not yet been clearly elucidated. Here we report the *in vivo* interaction of the endoplasmic reticulum (ER) chaperone BiP3 with the rice XA21 PRR, which confers resistance to the Gram negative bacterium, *Xanthomonas oryzae* pv. *oryzae* (*Xoo*). We show that XA21 is glycosylated and is primarily localized to the ER and also to the plasma membrane (PM). In BiP3-overexpressing rice plants, XA21-mediated immunity is compromised, XA21 stability is significantly decreased, and XA21 proteolytic cleavage is inhibited. BiP3 overexpression does not affect the general rice defense response, cell death or brassinolide*-*induced responses. These results indicate that BiP3 regulates XA21 protein stability and processing and that this regulation is critical for resistance to *Xoo*.

## Introduction

The innate immune response relies on recognition of pathogen-associated or microbe-associated molecular patterns (PAMPs or MAMPs) via a set of defined receptors known as pattern recognition receptors (PRRs) [1].

Plant and animal PRRs share conserved domains, such as leucine-rich repeats (LRRs) necessary for PAMP recognition [2], [3] and non-RD serine/threonine kinase domains that are either integral to the receptor (plants) or associated with it (animals) [4]. In animals, 13 TLRs have now been described [5]. All recognize PAMPs present in invading microorganisms and activate TLR-mediated signaling pathway [6]. In rice, XA21 recognizes a sulfated peptide, called Ax21 (activator of XA21-mediated immunity), present in all *Xanthomonas* and *Xylella* species [7], [8], [9]. In *Arabidopsis*, two additional plant PRRs have been identified and extensively characterized. These are *Arabidopsis* flagellin sensitive 2 (FLS2) and *Arabidopsis* elongation factor (EF)-Tu receptor (EFR). FLS2 and EFR recognize the flg22 peptide from flagellated bacteria and the EF-Tu-derived peptide elf18, respectively [10], [11].

Although PRRs are clearly essential for innate immunity in both animals and plants, a sustained or highly induced immune response can be harmful. Recent evidence suggests that dysregulated or impaired TLRs may lead to non-pathogenic diseases, such as chronic inflammation, autoimmune diseases, or cancer [12]. Similarly, improperly regulated plant immune responses can lead to the overexpression of defense-related genes and cell death [13], [14]. It is therefore necessary that the PRR signaling components, as well as the PRRs themselves, are tightly regulated. In *Arabidopsis*, FLS2 is negatively regulated by the kinase-associated protein phosphatase, KAPP, which blocks activated FLS2 signaling and attenuates the downstream innate immune response [15]. XA21 also recruits a protein phosphatase 2C, XB15, to attenuate XA21 signaling [13], [16].

In animals, extracellular PRRs are translated on the ER membrane, enter the ER lumen, and then are transported to the PM [17]. Despite fifteen years of research on PRR-mediated signaling, it is unclear how PRRs are processed and transported. The mechanism of release to the PM after translocation into the ER has not yet been elucidated. Nor is it known what types of signals are responsible for transportation from the ER. To date, ER processing of PRRs has not been demonstrated in plants. Recently a dedicated subset of ER-QC components is specifically required for the proper accumulation of a subset of PRRs has been demonstrated [18].

BiP, an abundant heat shock protein (HSP) 70 in the ER, is a multifunctional protein. It activates an adaptive signaling pathway termed the “unfolded protein response” that is fundamental to the health and development of human cells, organs and tissues [19]. BiP's intrinsic adenosine triphosphatase (ATPase) activity regulates binding and release from its substrates. In many animal systems, BiP interacts with the growing nascent chain of substrates containing N-linked glycans, facilitating their translocation into the ER [20]. In addition, it is involved in the quality control (QC) system by which misfolded or unassembled proteins are selectively retained in the ER [19]. BiP also targets permanently misfolded proteins for ER-associated degradation (ERAD) in mammals and yeast [19].

Here we report that rice BiP3 (also known as glucose-regulated protein 78; GRP78) interacts with rice XA21 *in vivo* and interfere XA21-mediated immunity.

## Results

### BiP3 Interacts with XA21 *in Planta*

**Figure 1:**
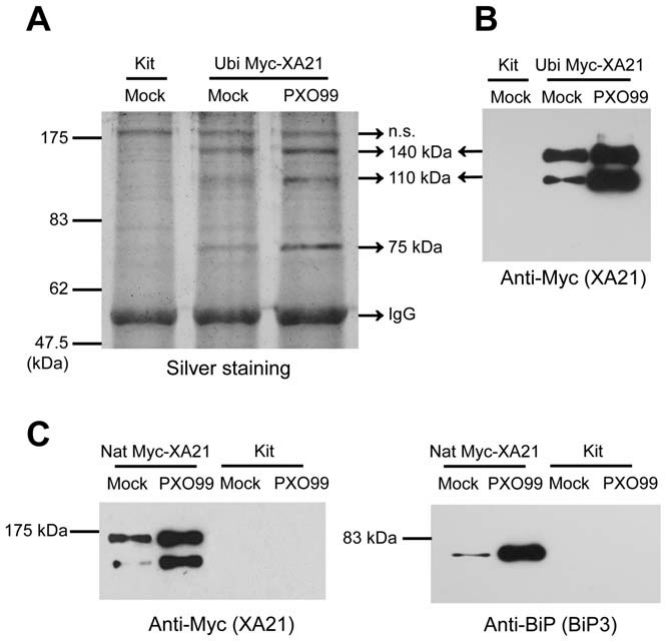
Rice BiP3 Interacts with XA21 *In Vivo*. (A) An XA21 complex was isolated from Ubi Myc-XA21 transgenic rice after *Xoo* strain PXO99Az inoculation. Five grams of leaves from Ubi Myc-XA21 or Kit were treated with *Xoo* or water for 12 h. After separation by SDS-PAGE, co-immunoprecipitated proteins were detected by silver staining. A 75 kDa protein co-immunoprecipitated with the XA21 protein. (B) XA21 was detected after co-immunoprecipitation. Myc-XA21 and Myc-XA21^cp^ displayed bands at about 140 and 100 kDa, respectively, as reported previously [16], [21]. (C) BiP3 co-immunoprecipitated with XA21 before (Mock) and after *Xoo* strain PXO99Az inoculation (*Xoo*) in transgenic rice carrying *Myc-Xa21* under the control of its native promoter. The precipitates were used for protein gel blot analysis using anti-Myc antibody (left) or anti-BiP antibody (right). Myc-XA21 and Myc-XA21^cp^ displayed bands at about 140 and 100 kDa, respectively, and BiP3 was detected as a 75 kDa band.

To identify components of XA21-mediated immunity, we isolated an *in vivo* XA21 protein complex using transgenic plants carrying an N-terminal Myc epitope-tagged *Xa21* gene under the control of the ubiquitin promoter (Ubi Myc-XA21). The transgenic Ubi Myc-XA21 plants were fully resistant to *Xoo* strain PXO99Az possessing Ax21 (Figure S1). We used an agarose-conjugated anti-Myc antibody to immunoprecipitate the XA21 complex before and after PXO99Az inoculation, and visualized the co-immunoprecipitated proteins by SDS-PAGE and silver staining (Figure 1A). We previously reported that the 140 kDa polypeptide is Myc-XA21 and the 110 kDa polypeptide is a proteolytic cleavage product of Myc-XA21 (Myc-XA21^cp^) by western blot analysis using anti-Myc antibody (Figure 1B) [16], [21]. In addition to the 140 and 110 kDa Myc-XA21 proteins (Figure 1B), we identified an approximately 75 kDa protein that accumulated after *Xoo* strain PXO99Az treatment (Figure 1A). The 75 kDa protein was not co-immunoprecipitated in the Kitaake (Kit) control plant lacking *Myc-Xa21*.

We sequenced the unknown 75 kDa protein as well as the putative XA21 and XA21^cp^ proteins using liquid chromatography-mass spectrometry/mass spectrometry (LC-MS/MS). Over thirty peptides generated from the 75 kDa protein matched “BiP3 (Os02g02410)”, one of five members of the BiP subfamily present in the rice genome (Figure S2A). Figure S3A shows the phylogenetic relationship of BiP proteins from human, yeast, *Arabidopsis*, and rice. Rice BiP3 (OsBiP3) shows the greatest similarity to *Arabidopsis* BiP1 and BiP2 with 89.2% and 89.3% identity, respectively. All peptides generated from the 140 kDa proteins matched XA21 from the N-terminal LRR region to the C-terminal kinase domain. In contrast, the peptides generated from the 110 kDa protein matched the LRR, but not the kinase domain (Figure S2B). These results indicate that XA21 proteolytic cleavage occurs between the LRR and the kinase domain, as previously predicted [16], [21].

To further investigate the association between XA21 and BiP3 *in vivo*, we generated transgenic plants carrying *Myc-Xa21* under the control of its native promoter (Nat Myc-XA21). The transgenic Nat Myc-XA21 plants were fully resistant to *Xoo* strain PXO99Az [13]. When the Myc-XA21 protein was immunoprecipitated with an agarose-conjugated anti-Myc antibody, the 140 kDa and 110 kDa polypeptides were detected (Figure 1C, left panel). Although the same amount of total protein extract was used for each immunoprecipitation, the Myc-XA21 protein precipitated with the anti-Myc antibody accumulated to greater amounts 12 hours (h) after *Xoo* strain PXO99Az inoculation, as compared to mock-treated Myc-XA21.

To examine the presence of BiP3 protein in the XA21 complex, we used a commercial anti-BiP antibody raised against amino acids 541 to 635 near the C-terminus of *Arabidopsis* BiP1 [22]. According to a Gene Expression Evidence Search (http://www.tigr.org/tdb/e2k1/osa1/locus_expression_evidence.shtml), BiP3 is the dominant form among rice BiPs. Because the peptide sequence of *Arabidopsis* is highly conserved in rice BiP3 (showing over 85% identity, in contrast to less than 60% identity with the rest of the BiP family members), we hypothesized that the anti-BiP antibody should successfully detect rice BiP3 (Figure S3B). Indeed, the anti-BiP antibody produced only one band, corresponding to a 75 kDa polypeptide in Kit plants (data not shown). We next examined whether BiP3 co-immunoprecipitated with XA21 using the anti-BiP antibody. The association between BiP3 and XA21 was detectable before *Xoo* strain PXO99Az treatment, and significantly increased 12 h after treatment (Figure 1C, right panel). In a control experiment, we detected no interaction between BiP3 and the Myc peptide (data not shown). These results demonstrate an *in vivo* interaction between BiP3 and XA21.

### BiP3 Possesses ATPase Activity

The gene encoding BiP3 has a 1,998 bp open reading frame that consists of seven introns and eight exons. It is predicted to encode a 666 amino acid protein with a molecular mass of 73.4 kDa and an isoelectric point of 5.0. BiP3 is similar in overall structure to other known HSP70s in plants and animals, with an approximately 45 kDa domain at the N-terminus that is predicted to carry ATPase catalytic activity and a domain of approximately 25 kDa at the C-terminus having a predicted substrate-binding domain [23].

To examine whether the predicted N-terminal ATPase domain of BiP3 has enzymatic activity, full-length BiP3 was expressed as an N-terminal tagged Glutathione-S-transferase (GST) recombinant fusion protein (GST-BiP3) in *Escherichia coli* (*E*. *coli*). We purified and assayed the recombinant protein for ATPase activity by a standard method, measuring the release of phosphate from ATP in the presence of 5 mM MgCl_2_ (Figure S4A) [24]. The recombinant fusion protein, GST-BiP3, demonstrated ATPase activity, but purified GST alone or boiled GST-BiP3 did not (Figure S4B), indicating that *BiP3* encodes a functional ATPase.

### Overexpression of *BiP3* Compromises XA21-Mediated Immunity

To investigate the biological relevance of BiP3 in XA21-mediated immunity, we first generated a transgenic line expressing *Xa21* under control of its native promoter (Nat XA21). The transgenic Nat XA21 plants were fully resistant to *Xoo* strain PXO99Az (Figure S1). We then generated double transgenic rice overexpressing *BiP3* (BiP3 ox) under the control of the ubiquitin promoter in the Nat XA21 genetic background. Fourteen double transgenic lines (BiP3 ox/Nat XA21) were assayed for overexpression of *BiP3* using the anti-BiP antibody. In all 14 BiP3 ox/Nat XA21 lines, there was a significant increase in BiP3 protein compared to the Kit or XA21 control (data not shown). All BiP3 ox/Nat XA21 lines demonstrated normal growth and development compared to the Nat XA21 and Kit control plants.

**Figure 2:**
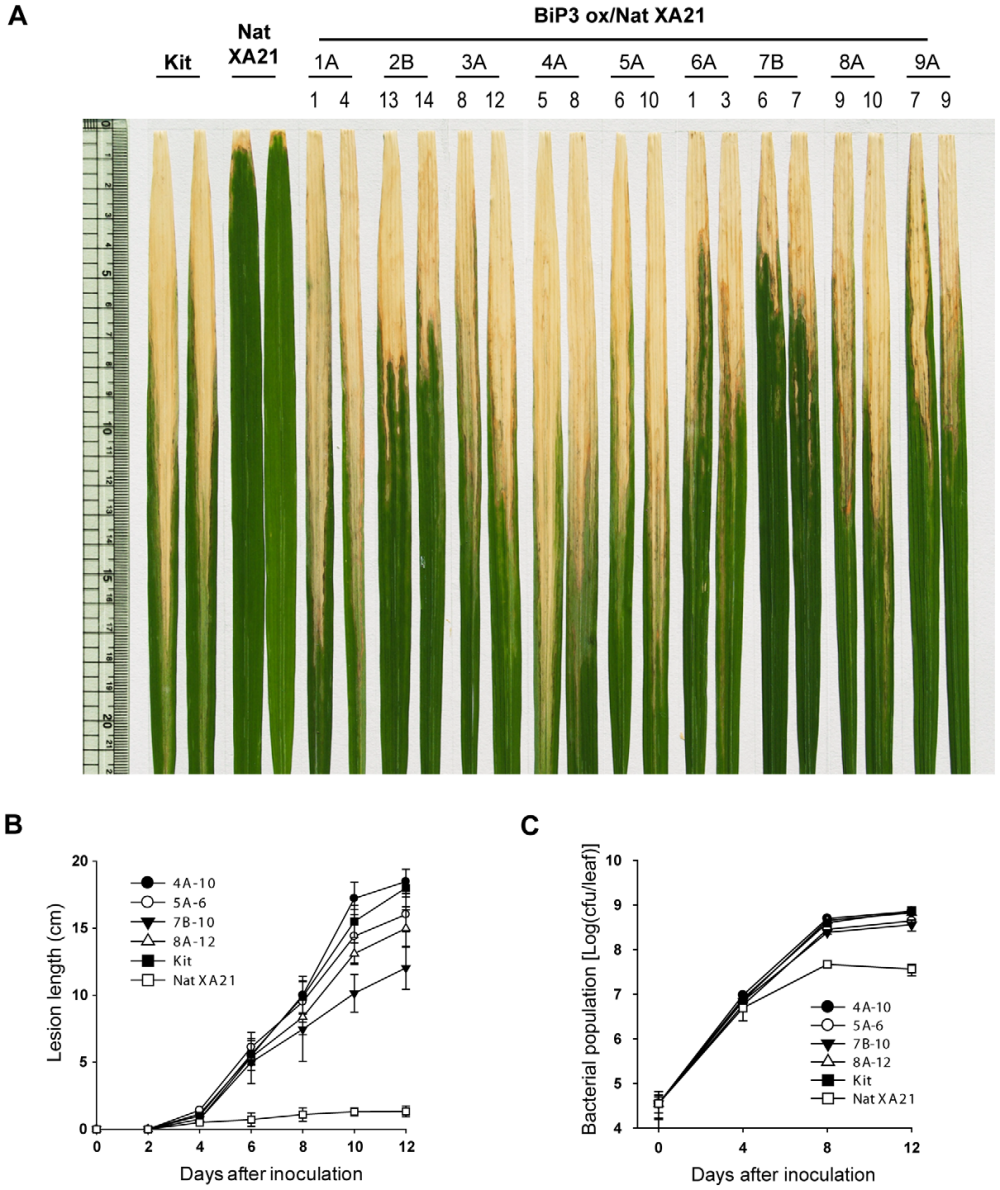
Overexpression of *BiP3* Compromises XA21-Mediated Resistance. (A) Rice lines 14 days after inoculation with *Xoo* strain PXO99Az. From left to right: Kitaake (Kit), transgenic line (Nat XA21) carrying *Xa21* driven from its native promoter, and transgenic lines carrying *BiP3 ox* (BiP3 ox/Nat XA21). (B) Lesion length measurements of *Xoo* strain PXO99Az inoculated plants, BiP3 ox/Nat XA21 (4A-10, 5A-6, 7B-10, and 8A-12), Kitaake (Kit), and Nat XA21 control lines over 12 days. Each data point represents the average and standard deviation of at least four samples. (C) *Xoo* strain PXO99Az populations were monitored over 12 days in BiP3 ox/Nat XA21 (4A-10, 5A-6, 7B-10, and 8A-12), Kit, and Nat XA21 rice lines. For each time point, bacterial populations were determined in three separate leaves for each genotype. Capped vertical bars represent standard deviation values (cfu/leaf) obtained from the three samples.

We then analyzed the double transgenic lines, BiP3 ox/Nat XA21 (T_0_ generation), at 6 weeks of age for alterations in resistance to *Xoo* strain PXO99Az. All 14 independently transformed XA21 lines overexpressing *BiP3* either completely or partially lost XA21-mediated resistance and displayed significantly enhanced susceptibility to *Xoo* strain PXO99Az compared to the control transgenic plants carrying *Xa21* alone (data not shown). To confirm that the observed phenotype in BiP3 ox/Nat XA21 (T_0_) was due to the *BiP3* transgene, T_1_ progeny were analyzed. BiP3 ox/Nat XA21 (T_1_) plants maintained a high accumulation of BiP3 protein, similar to the levels observed in the T_0_ parent (Figure S5). In figure 2A, we show two typical leaves from each of the inoculated rice plants: wild type Kit, Nat XA21 control, and BiP3 ox/Nat XA21 at 14 days after *Xoo* strain PXO99Az inoculation. While the Nat XA21 control was highly resistant, showing short lesions (approximately 1 to 2 cm), the inoculated leaves of the double transgenic lines (BiP3 ox/Nat XA21) developed water-soaked, long lesions (approximately 15 to 20 cm) typical of bacterial blight disease. Segregants from the double transgenic lines carrying *Xa21* but lacking *BiP3 ox* showed no significant differences in lesion lengths as compared to the Nat XA21 plants (data not shown).

To quantify the effect of BiP3 overexpression, the BiP3 ox/Nat XA21 plants (T_1_) were inoculated with *Xoo* PXO99Az and lesion lengths and bacterial growth were monitored over time (Figures 2B and 2C). At four days after inoculation (DAI), slight increases in the lesion lengths and bacterial population were detected in the BiP3 ox/Nat XA21 lines (4A-10, 5A-6, 7B-10, and 8A-12) compared with the Nat XA21 control (Figure 2B). At 12 DAI, the double transgenic rice BiP3 ox/Nat XA21 displayed significantly enhanced susceptibility to *Xoo* strain PXO99Az, with lesions ranging in length from 15 to 20 cm compared to the Nat XA21 control, which displayed lesion lengths of 1 to 2 cm. The bacterial growth curve correlated well with lesion length developments (Figure 2C). *Xoo* strain PXO99Az populations in Nat XA21 transgenic rice reached approximately 4.75×10^7^ colony-forming units per leaf (cfu/leaf), whereas the population in Kitaake (Kit) plants reached to more than 8.85×10^8^ cfu/leaf. In BiP ox/Nat XA21 line 4A-10, *Xoo* strain PXO99Az populations grew to 7.90×10^8^ cfu/leaf, a greater than sixteen-fold increase compared to the Nat XA21 control. The BiP3 ox/Nat XA21 lines, 4A (4A-4) and 5A (5A-9), which harbored the highest levels of BiP3 protein accumulation (Figure S5), displayed the longest lesions and the largest bacterial populations of all lines tested, indicating that XA21-mediated immunity is regulated by BiP3 in a dosage-dependent manner. These results demonstrate that overexpression of BiP3 reduces XA21-mediated resistance.

### Silencing of *BiP3* Does Not Affect XA21-Mediated Immunity

To further investigate the role of BiP3 in XA21-mediated immunity, transgenic rice line silenced for *BiP3* (BiP3 RNAi) were generated (Figure S6A) and BiP3 RNAi 5A line was crossed with transgenic Kitaake lines (pollen recipient) possessing either *Myc-Xa21* under the control of *Ubi* or its native promoter (Ubi Myc-XA21 and Nat Myc-XA21, respectively). The presence of *Myc-Xa21* and/or *BiP3 RNAi* in the F_1_ progeny was confirmed by PCR analysis (data not shown). We then inoculated the F_1_ progeny with *Xoo* strain PXO99Az. We found no difference in the response of Myc-XA21×BiP3 RNAi lines to *Xoo* strain PXO99Az in terms of lesion lengths as compared to Kitaake and BiP3 RNAi transgenic lines (Figure S6B). This result indicates that although accumulated BiP3 protein inhibits XA21-mediated immunity, silencing of BiP3 does not result in an observable phenotype.

To test the hypothesis that another BiP family member serves a redundant function in Myc-XA21×BiP3 RNAi plants, we analyzed the expression of six BiP subfamily members using a Gene Expression Evidence Search (http://www.tigr.org/tdb/e2k1/osa1/locus_expression_evidence.shtml). Although *BiP3* is the most highly expressed BiP gene family member, a closely related BiP member, called BiP5, is also expressed. We found that *BiP5* gene expression levels are moderately enhanced in the BiP3 RNAi lines (3A-1 and 5A-2) (Figure S6C). This result suggests that *BiP5* expression may compensate for the lack of BiP3 in the *BiP3* silenced lines. Such a functional redundancy would explain the lack of observable phenotype in *BiP3* RNAi lines. To further test this hypothesis, we investigated if BiP5 could also interact with XA21. We carried out another coimmunoprecipitation experiment using increased amounts of rice leaf tissue. After LC/MS-MS analysis, we found four BiP5-specific peptides, which indicate that BiP5 is also a component of XA21 complex (Figure S6D).

Despite numerous attempts, we were not able to obtain any transgenic plants silencing both BiP3 and BiP5 in wild-type or XA21 genetic backgrounds. This result is not surprising because it has previously been shown that BiP is an essential gene for viability of *Saccharomyces cerevisiae* [25] and mice [26], [27]. In tobacco and Arabidopsis, a reduction in the basal level of BiP members is deleterious to cell viability [28], [29].

### Alteration in *BiP3* Expression Does Not Affect the General Rice Defense Response or Cell Death

To determine if altered BiP3 can affect the general defense response, we generated BiP3 ox and BiP3 RNAi transgenic rice lines lacking *Xa21*. After confirming that *BiP3* is overexpressed or silenced in the transgenic lines by RT-PCR with *BiP3* specific primers (data not shown), we inoculated BiP3 ox and BiP3 RNAi transgenic rice lines with PXO99Az. We found no difference in lesion lengths or bacterial multiplication in the transgenic lines compared to wild type Kitaake (data not shown). In addition, these transgenic rice lines did not display any obvious cell death phenotype in the presence or absence of pathogen. These results indicate that alterations of *BiP3* expression levels in the absence of *Xa21* do not affect the defense response or cell death.

### XA21 and BiP3 Are Localized to the ER

To elucidate the *in vivo* function of BiP3 regulation of XA21, we investigated the cellular distribution of BiP3 and XA21. An *in vivo* targeting experiment was performed using fusion proteins with smGFP2 as the fluorescent marker [30]. The *Ubi smGFP2*, *Ubi BiP3-smGFP2*, or *Ubi Xa21-smGFP2* constructs were introduced into rice protoplasts by PEG−mediated transformation [31] (Figure S7A). The localization of the fusion proteins was determined by visualization with a confocal microscope. The smGFP2 control was uniformly distributed throughout the rice protoplasts, including the nucleus. BiP has been previously shown to be ER localized in animal and plant cells [32], [33], [34]. As expected, the BiP3-smGFP2 fusion protein was mainly localized to the ER, supporting a functional role for BiP3 as a chaperone in the ER. The XA21-smGFP2 fusion protein was also mainly localized to the ER of protoplasts, which is consistent with our finding that XA21 co-immunoprecipitates with an ER chaperone, BiP3, *in vivo*. Figure S7B shows confocal images obtained from an identical cell, but focused on the ER (left) and PM (right), respectively. As a positive control for the PM, we used N-(3 triethylammoniumpropyl)-4-(6-(4(diethylamino) phenyl) hexatrienyl) pyridinium dibromide (FM4-64), which stains the PM of protoplast cells. A close overlap was observed between XA21-smGFP2 and FM4-64, suggesting that XA21 may also be partly localized to the PM, which fits our previously published results indicating that Ax21 recognition occurs at the PM [7]. However at the resolution used, it was not possible to conclusively distinguish the PM from the ER.

**Figure 3:**
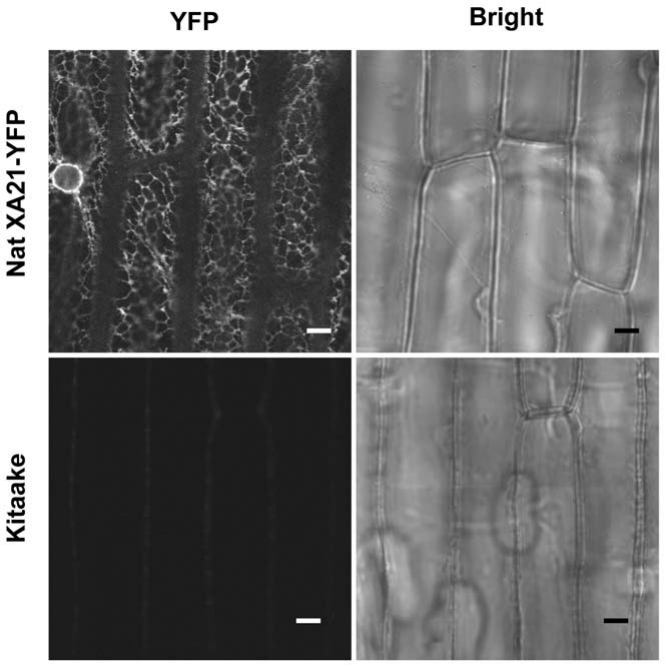
XA21 Is Mainly Localized to the Endoplasmic Reticulum in Rice Leaf Sheath Tissue. *In planta* subcellular localization of Nat XA21-YFP (top) and non-transgenic Kitaake (bottom) were determined using confocal microscopy. Intact adaxial sheath epidermal cells were imaged with an Olympus FV1000 confocal microscope equipped with a 60×oil immersion lens [numerical aperture, 1.42]. YFP signal was excited at 515 nm and emission was collected between 530-560 nm. Scale bar, 5 µm.

To confirm the ER accumulation of XA21 and rule out the possibility that the accumulation was caused by overexpression of XA21, we generated transgenic plants containing *Xa21-YFP* under the control of its native promoter (Nat XA21-YFP). These lines express the full-length XA21-YFP fusion protein as detected by reaction with anti-GFP antibody (data not shown) and are fully resistant to *Xoo* strain PXO99Az (Figure S8). These results indicate that the Nat XA21-YFP fusion protein is biologically equivalent to the native XA21 protein, making this line suitable for localization studies. For confocal imaging, we utilized rice leaf sheath epidermal cells from transgenic plants. This tissue is optically clear and relatively flat, which facilitates live-cell imaging [35]. Figure 3 shows that Nat XA21-YFP fusion protein is localized mainly to a reticular structure characteristic of the cortical ER and a perinuclear region of sheath epidermal cells, indicating that XA21 accumulates in the ER of intact plant cells. Control plants constitutively expressing YFP from the Ubi promoter (Ubi YFP) showed strong YFP signal in the cytoplasm and nucleus (data not shown), consistent with previous reports [30].

### XA21 Is a Glycosylated Protein

The majority of the PM proteins synthesized in the ER undergo glycosylation [36]. In animals, many PRRs including TLR4 and 9, require N-linked glycosylation for receptor function [37]. *Arabidopsis* FLS2 and rice XA21 are also predicted to be extensively glycosylated [11], [38]; however, this has not yet been experimentally proven. For example, the molecular mass of XA21 isolated from rice plants is significantly larger (approximately 140 kDa) than that predicted based on its primary amino acid sequence (approximately 110 kDa) [16], [21].

**Figure 4:**
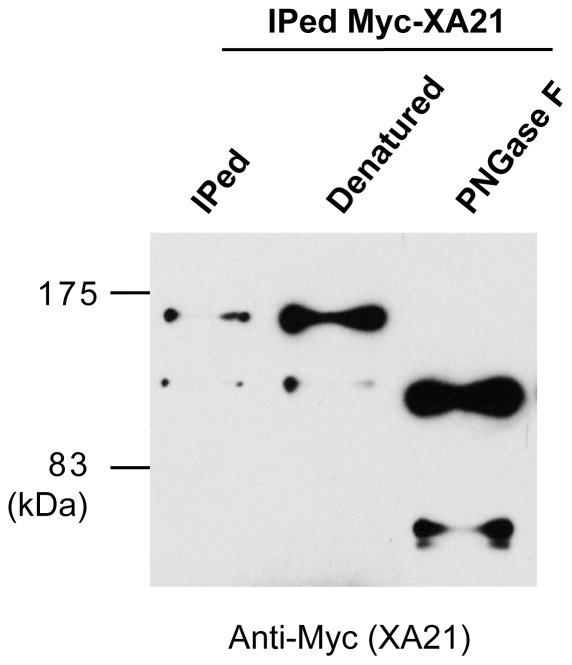
XA21 Is a Glycosylated Protein. Myc-XA21 protein was immunoprecipitated from Ubi Myc-XA21 rice using anti-Myc antibody. After washing the Myc-XA21 protein bound to anti-Myc antibody-conjugated agarose beads, Myc-XA21 protein was digested with PNGase F for 2 h at 37°C. Immunoprecipitated Myc-XA21 (IPed) was loaded in the first lane. IPed Myc-XA21 was denatured (second lane) and then treated with PNGase F (third lane). The samples were then subjected to SDS-PAGE for western blot analysis with anti-Myc antibody.

To investigate whether XA21 is glycosylated, we treated the protein with the peptide-N-glycosidase F (PNGase F) enzyme, which removes all glycosyl groups [39], and evaluated the mobility of treated and untreated Myc-XA21 by SDS-PAGE. After immunoprecipitation with anti-Myc antibody, full-length Myc-XA21 and the putative cleavage product, Myc-XA21^cp^, migrate as bands of approximately 140 kDa and 110 kDa, respectively (IPed) (Figure 4). After digestion of denatured Myc-XA21 with PNGase F, we observed a major change in the mobility of Myc-XA21 and Myc-XA21^cp^, with bands of approximately 110 kDa and 70 kDa, respectively. These results reveal that XA21 is highly glycosylated, which is responsible for its retardation in SDS-PAGE.

### Transgenic Rice Plants Overexpressing *BiP3* Fail to Accumulate and Process XA21

Yeast and animal BiPs are involved in targeting unfolded glycoproteins for ERAD machinery [19]. If glycoproteins are not able to acquire their native conformations within an appropriate time, terminally misfolded proteins are retained due to the QC system present in the ER and ultimately destroyed by ERAD [19], [40]. We hypothesized, therefore, that accumulation of XA21 in the BiP3-overexpressing plants may be affected by the ERAD as a QC mechanism.

**Figure 5:**
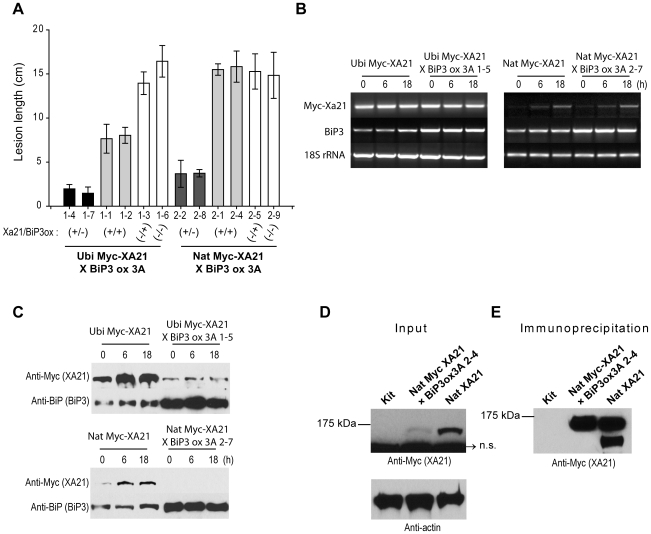
Transgenic Lines Overexpressing *BiP3* Fail to Accumulate the XA21 and Its Cleavage Product. (A) Lesion length measurements of F_2_ population segregating for Myc-XA21 and overexpressed BiP3 (BiP3 ox). The F_2_ segregants (*Xa21*/*BiP3ox*; +/−, +/+, −/+, −/−) were inoculated with *Xoo* strain PXO99Az and lesion lengths were measured 12 days post-inoculation. Nat Myc-XA21: *Xa21* driven by the native promoter. Ubi Myc-XA21: *Xa21* driven by the maize ubiquitin promoter. (B) RNA accumulation of the *Myc-Xa21* and *BiP3* transcripts in F_2_ segregants 3A 1–5 and 3A 2–7 before and after *Xoo* strain PXO99Az inoculation. Lines are as described in (A). Total RNA was extracted and RT-PCR was performed using *Myc-Xa21* and *BiP3*-specific primers. Control RT-PCR reactions were carried out with *18S rRNA*-specific primers. (C) Protein accumulation of the Myc-XA21 and BiP3 in F_2_ segregants 1–5 and 1–7 before and after *Xoo* strain PXO99Az inoculation. Equal amounts (100 µg) of total protein form Ubi Myc-XA21, Ubi Myc-XA21×BiP3ox3A 1-5, Nat Myc-XA21, and Nat Myc-XA21×BiP3ox3A 1–7 were extracted after *Xoo* strain PXO99Az inoculation, analyzed by SDS-PAGE, and immunoblotted with anti-Myc and anti-BiP antibodies. (D) Total protein was extracted from Kit, Nat Myc-XA21/BiP3 ox, and Nat Myc-XA21 plants after *Xoo* strain PXO99Az inoculation. Equal amounts (300 µg) of total protein were analyzed by SDS-PAGE and immunobloted with anti-Myc antibody. Equal total protein loading was confirmed with anti-actin antibody. A nonspecific band (n.s.) of 95 kD was detected. (E) After immunoprecipitation with anti-Myc antibody, western blot analysis was performed to visualize XA21 and its cleavage product. Immunoprecipitates from Nat Myc-XA21 were diluted ten-fold, resulting in similar amounts of XA21 in Nat Myc-XA21/BiP3 ox and in Nat Myc-XA21.

To test the hypothesis, transgenic Kitaake lines (pollen recipient) possessing either *Myc-Xa21* under the control of *Ubi* or its native promoter (Ubi Myc-XA21 and Nat Myc-XA21, respectively) were crossed with another transgenic rice line overexpressing *BiP3* (BiP3 ox 3A, pollen donor). The presence of *Myc-Xa21* and/or *BiP3 ox* in the F_2_ progeny was confirmed by PCR analysis (data not shown). The F_2_ progenies were inoculated with *Xoo* strain PXO99Az and examined for cosegregation of the genotype with the disease phenotype (Figure 5A). All *Nat Myc-Xa21* plants overexpressing *BiP3* (Nat Myc-XA21×BiP3 ox, +/+) displayed significantly enhanced susceptibility to *Xoo* strain PXO99Az, displaying long lesions (approximately 15.5 cm) comparable to those of the segregants lacking *Nat Myc-Xa21* (−/− and −/+). In contrast, *Ubi Myc-Xa21* plants overexpressing *BiP3* (Ubi Myc-XA21×BiP3 ox, +/+) displayed partial resistance, with shorter lesions (approximately 7.5 cm) compared to the Nat Myc-XA21×BiP3 ox plants (+/+). These results suggest that the observed enhanced susceptibility in the BiP3-overexpressing lines is due to altered amounts of XA21 protein.

We next examined the transcripts and protein accumulation of *Myc-Xa21* and *BiP3* in the F_2_ progeny (Figures 5B and 5C). In the Ubi Myc-XA21 line, a slight accumulation of endogenous *BiP3* was observed after *Xoo* inoculation (Figure 5B). In contrast, significant amounts of *BiP3* transcripts and their corresponding proteins were detected in the F_2_ line, Ubi Myc-XA21×BiP3 ox 3A 1-5, suggesting that the *BiP3* transgene is constitutively overexpressed, regardless of *Xoo* inoculation. *Myc-Xa21* under the control of the *Ubi* promoter was also expressed constitutively. The Myc-XA21 protein accumulated to high levels before *Xoo* treatment and was significantly induced after inoculation of the Ubi Myc-XA21 line (Figure 5C). However, in the F_2_, the protein accumulated to much lower levels compared to Ubi Myc-XA21, with very little further accumulation after *Xoo* inoculation. Inhibition of XA21 accumulation was also observed in the BiP3 ox line containing *Nat Myc-Xa21*. In the F_2_, Nat Myc-XA21×BiP3 ox 3A 1–7, XA21 was barely detected regardless of *Xoo* treatments, in congruence with the susceptible phenotype observed in Figure 5A. Equal total protein loading was confirmed using anti-actin antibody (data not shown).

We next investigated whether XA21 processing was affected by BiP3 overexpression. After immunoprecipitation of XA21 from Nat Myc-XA21×BiP3 ox and Nat Myc-XA21 plant, the amounts of XA21 and its cleavage product, XA21^cp^, were compared in both lines (Figure 5D). The 140 kDa of full-length XA21 and the 110 kDa protein representing the cleaved product, respectively, were detected in Nat Myc-XA21. In contrast, although a concentrated immunoprecipitate of the F_2_ line was used and the 140 kDa full-length XA21 protein was detected, XA21^cp^ was not (Figure 5E). Taken together, these results indicate that the accumulation and processing of XA21 after *Xoo* inoculation is significantly inhibited by overexpression of the ER chaperone BiP3 and suggest that continuous and/or prolonged binding of overexpressed BiP3 results in XA21 degradation possibly via the ERAD.

### Overexpressed BiP3 Does Not Affect Brassinolide-Induced Responses

We have shown that XA21 is degraded in BiP3 ox lines (Figure 5). Based on this result, we hypothesized that overexpressed BiP3 may affect the stability of other receptor kinases (RKs) and therefore interfere with their signal pathways. We therefore tested if BiP3 affects brassinolide (BL)-induced responses mediated by the *brassinosteroid insensitive 1* (*OsBRI1*) RK.

Like *Xa21*, *BRI1* encodes a RK that has an extracellular domain containing LRRs [41]. Unlike XA21, however, OsBRI1 carries an RD kinase domain [4]. RD RKs are generally not associated with pathogen recognition in the absence of complexes formed with non-RD RKs [4], [42]. Whereas wild-type *OsBRI1* rice lines show elongated coleoptiles and reduced root elongation when grown in BL, mutants that are disrupted in *OsBRI1*-mediated signaling are not affected by BL treatments [43].

**Figure 6:**
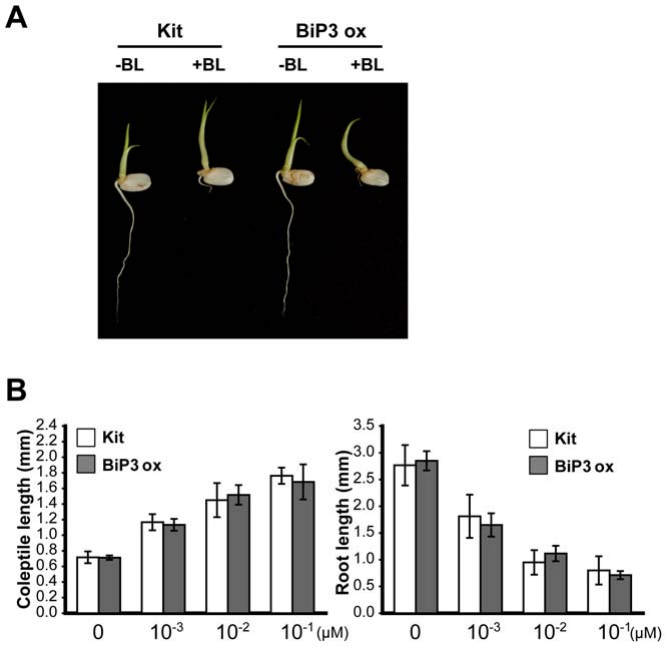
BiP3 Does Not Interfere with Brassinolid-Induced Responses. (A) Seeds from Kitaake (Kit) and the BiP3 ox 3A line (BiP3ox) were germinated on MS agar in the presence (+) or absence (−) of 0.1 µM BL. Seedlings were examined 3 days after germination. (B) Effect of BL on coleoptile and root elongation in Kit and BiP3 ox seedlings. The plants were germinated in MS agar plates containing the indicated concentration of BL. Data presented are the means of results from four plants. Bars indicate SD.

We hypothesized that if BiP3 negatively regulate the stability of OsBRI1, then BiP3 ox lines would show alterations in response to BL. We therefore germinated wild-type rice plants (Kitaake) on 0.1 µM BL plates. We found that, the coleoptiles elongated abnormally and root elongation was inhibited (Figure 6). We next tested the effects of BL on BiP3 ox 3A lines germinated on BL plates. We found that these lines displayed a wild-type phenotype, with inhibited root growth and elongated coleoptiles (Figure 6A). To quantify these effects, we measured the effects of a range of BL concentrations on the length of coleoptiles and roots of seedlings (Figure 6B). In both Kitaake and the BiP3 ox lines, the coleoptiles were longer and the root lengths were shorter after treatment with BL. In adult plants, the BiP3 ox line did not display a dwarf phenotype characteristic of the *OsBRI1* mutant lines [43] (data not shown). XA21 and BiP3 ox/XA21 3A-3 lines germinated in BL plates displayed the same phenotype to Kitaake and BiP3 ox lines (Figure S9). Taken together, these results indicate that alteration in BiP3 protein expression does not interfere with BRI1-mediated signaling and suggest that BiP3 specifically affects the XA21-mediated response. Li and coworkers showed that Arabidopsis BiPs did not interact with wild-type *Arabidopsis* BRI1 [29], supporting the conclusion that the BiP ER chaperones can be quite specific to it substrates.

## Discussion

Rice XA21 is representative of a very large class of plant cell surface receptors (371 in rice and 47 in *Arabidopsis*) predicted to function in innate immunity [4]. In this study, we show that BiP3, an ER-localized chaperone HSP70, regulates XA21 processing and stability. Although there are several ER-localized HSPs that can potentially chaperone PRRs, including calnexin, calreticulin, protein disulfide isomerase, gp96, thiol-oxidoreductase ERp57, and BiP, to date only gp96 has been demonstrated to function in PRR-folding [44]. Our results now show that BiP can also serve as a PRR chaperone, and that it is involved in processing and degradation of XA21.

In animals, BiPs have been shown to interact with various cell surface proteins such as γ-aminobutyric acid type A receptor, α-amino-3-hydroxy-5-methyl-4-isoxazolepropoinate (AMPA) receptor, nicotinic acetylcholine receptor, and epidermal growth factor receptor (EGFR) [32], [45]. Whereas overexpression of human BiP inhibits translocation of EGFR to the cell surface [32], it has no effect on AMPA receptor expression [46]. In plants, although a role for regulation of cell surface receptors by BiP has not previously been demonstrated, it has been shown that *Arabidopsis* BiP interacts with a mutant form of the brassinosteroid (BR) receptor, bri1-9, but not the wild-type BR receptor [29], [47]. These results suggest that *Arabidopsis* BiP prevents the export of a structurally perturbed BR receptor.

In the ER, increased protein synthesis followed by accumulation of unfolded and/or misfolded proteins cause “ER stress” [19]. BiP is induced during the ER stress and carries out its role in protein folding and assembly [19], [48]. In *Arabidopsis*, BiP2 is involved in folding and secretion of PR proteins during systemic acquired resistance (SAR) [22]. In a *bip2* mutant, increased PR protein synthesis after benzothiadiazole S-methylester (BTH, salicylic acid analog) treatment is not accompanied by a concomitant increase in BiP protein accumulation. This results in an intracellular accumulation of unfolded proteins in the ER [22].

**Figure 7:**
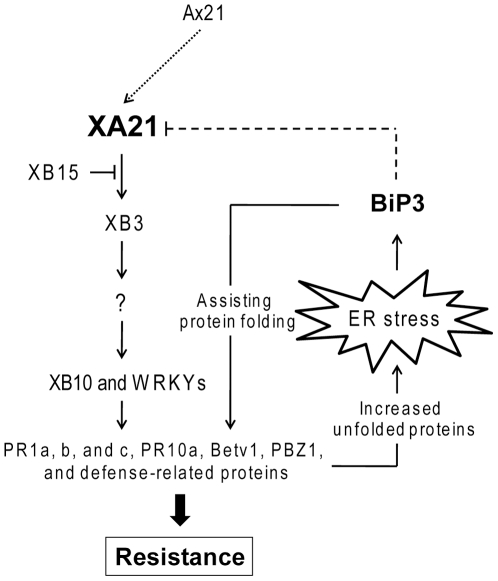
A Model for Regulation of XA21-Mediated Innate Immunity by BiP3. The XA21 LRR domain is responsible for recognition of *Xoo* strains carrying Ax21 [2], [7], [9]. XA21/Ax21 binding is hypothesized to activate the non-RD kinase domain leading to XA21 autophosphorylation and/or transphosphorylation of downstream target proteins [7], [13], [16]. XA21 transphosphorylates the RING finger ubiquitin ligase XB3, which is required for effective XA21-mediated resistance [16]. XB10 (OsWRKY62) [56] and other WRKY transcription factors either activate or repress PR genes [13], [56]. In plants, pathogen infection upregulates expression of PR genes [13], [57], [58], [59], resulting in increased translations of the corresponding proteins in the ER. The transiently accumulated unfolded and/or misfolded proteins cause ER stress, which, in *Arabidopsis*, activates *BiP*s [48]. During systemic acquired resistance, BiP helps to secrete accumulated PRs [22]. Our results suggest that excessive loading of unfolded and/or misfolded proteins during prolonged ER stress attenuates the signal transduction pathway causing the ER stress. In support of this model, BiP3 overexpression drives XA21 proteolysis and down-regulates the XA21-mediated immune response.

If the *in vivo* function of BiP3 is restricted to the secretion of PR proteins during SAR, then overexpression of BiP3 in the XA21 background would be expected to lead to enhanced resistance (Figure 7). Instead we found that that BiP3 ox/Nat XA21 double transgenic plants display compromised XA21-mediated resistance. These results indicate that in addition to functioning in SAR, BiPs can also inhibit cell surface receptor-mediated innate immunity. We hypothesize that if unfolded and/or misfolded proteins over-accumulate after *Xoo* infection, then ER stress will be prolonged. In this case, cells can either initiate ER-associated cell death or attenuate the signal transduction pathway causing the ER stress. Our results indicate that BiP3 attenuates the XA21-mediated signaling pathway. We also show that XA21, like TLR4 and TLR9, is highly glycoslyated. In the case of TLR4 and TLR9, N-glycosylation occurs in the ER during maturation [37]. This N-glycosylation is important for correct protein folding and ERAD [19], [40]. We, therefore, hypothesize that BiP3 accumulation drives glycosylated XA21 to the ERAD system, inhibiting its further processing (Figure 7). As a result, full-length XA21 protein and its cleavage product (XA21^cp^) are significantly reduced in transgenic plants overexpressing BiP3 (Figure 5).

Transgenic plants overexpressing BiP3 but lacking XA21 do not exhibit abnormal developmental or morphological phenotypes suggesting that the reduction in resistance observed when BiP3 is overexpressed is not a consequence of a general ER stress response. Transgenic lines overexpressing or silenced for *BiP3* also do not display cell death, which is often observed following severe ER stress in animals [49] and *Arabidopsis* [22]. To investigate if BiP3 overexpression affects signaling pathways mediated by other RKs, we investigated OsBRI1-mediated responses to brassinolide. Although OsBRI1 shows an overall structural similarity with XA21 [50], unlike XA21 it falls into the RD class of kinases. Thus these experiments allowed us to determine whether or not overexpression of BiP3 affected both non-RD and RD-mediated responses. We found thatBiP3 ox and BiP3 ox/XA21 lines are no less sensitive to low BL concentration (10^−3^ µM) than their controls, indicating that BiP3 overexpression does not interfere with OsBRI1-mediated signaling (Figure 6). Taken together, these results indicate that altered *BiP3* expression does not affect all RK-mediated signaling pathways and does not affect a general ER stress response. In support of our conclusion that ER chaperones can be quite specific to their substrates, it was recently shown that, despite of its role as a general housekeeping chaperone, gp96 is also specific for processing TLRs including TLR2, 4, 5, 7, and 9 in macrophages [44], [51]. For example, although gp96-deficient macrophages failed to respond to flagellin, the ligand for TLR5, mutant macrophages displayed normal development and activation by interferon-γ, tumor necrosis factor-α, and interleukin-1β [44].

Intramolecular cleavage of receptors, critical for their *in vivo* functions, has been observed in many receptors including EGFRs and TLRs. For example, human EGFR ErbB-4 is processed by two membrane-localized proteases [52]. The cleavage product, containing a tyrosine kinase domain, is translocated to the nucleus and is believed to phosphorylate nuclear substrates [52]. Mouse TLR9 is retained in the ER intracellularly [37] and cleaved in the endolysosome where ligand recognition occurs [53]. Although both full-length and processed forms of the receptor can bind the ligand, only the cleaved form of TLR is competent for signal transduction. Similarly, in rice, XA21D (a truncated form of XA21 lacking the transmembrane and cytoplasmic kinase domains), and XA21^K736E^ (a point mutant of XA21 lacking kinase catalytic activity) both recognize the presumed ligand, a sulfated peptide [2], [8], [54] and confer partial resistance to *Xoo* in transgenic plants. These results supports the hypothesis that alternate forms of the XA21 proteins are functional and suggest that XA21^cp^ may also serve a critical role in immunity rather than existing simply as an intermediate of cellular proteolysis. The fact that resistance conferred by XA21D and XA21^K736E^ is not as robust as that of XA21, indicates that the kinase domain is also critical for resistance, either as part of the intact receptor or as a cleavage product.

## Materials and Methods

### Plant Material and Growth Conditions

Rice (*Oryza sativa* L.) plants were maintained in the green house. The growth chamber was set on a 16 h light and 8 h dark photoperiod, a 28/26°C temperature cycle, and 90% humidity. Healthy and well-expanded leaves from 6-week-old rice plants were used for *Xoo* strain PXO99Az and nucleic acid or protein extraction.

### *Xoo* Inoculation and Determination of Bacterial Populations

For *Xoo* inoculation, rice plants were grown in the greenhouse normally until they were 6 weeks old, unless stated otherwise, and transferred to the growth chamber. The *Xoo* strain Philippine race 6 (*Xoo* strain PXO99Az) was used to inoculate rice by the scissors-dip method [38], [55]. Only the top two to three expanded leaves of each tiller were inoculated. For *Xoo* colony counts from inoculated leaves, 20 cm of leaf tissue from the top, including lesions and tissue showing no lesions, was ground up and resuspended in 10 ml water to harvest bacteria. The extract was diluted accordingly and plated out on peptone sucrose agar (PSA) plates containing 15 mg/l cephalexin.

### Plasmid Construction for *BiP3* Overexpression in Rice

A 1,998 nt cDNA fragment encoding full-length BiP3 protein was amplified from a rice cDNA using primers, 5′-CACCATGGATCGGGTTCGCGGATGCGCG-3′/5′-CTACAGCTCGTCATGCTCGTCGTCGAC-3′. The PCR product was cloned into pENTR^TM^/D-TOPO® (Invitrogen) according to the instructions provided by the manufacturer and the insert confirmed by sequencing. For over-expression in rice, the *BiP3* cDNA in pENTR^TM^/D-TOPO® was recombined into the final Ubi-pCAMBIA-1300 vector using Gateway® LR Clonase (Invitrogen). Construction of Ubi-CAMBIA-1300 has been described before[55]. To make it Gateway-compatible, a Gateway cassette was introduced into the multiple cloning sites located between the Ubi promoter and the Nos 3′ terminator.

### Construction of the *Ubi Myc-Xa21* and *Nat Xa21-YFP* Plasmids for Expression in Rice

To construct the *Ubi Myc-XA21* plasmid, a 5′ fragment of *Myc-Xa21* was PCR-amplified using primers, 5′-AAAGGATCCAACATCTCTCGCTGTCTT-3′/5′-GGCTGAGCTCCGGTGGTAT-3′ and template pC822-cMyc-Xa21. This 420-bp 5′-fragment was cut with BamHI/SacI at the ends and cloned, together with a 4.2-kb *Sac*I/*Spe*I *Xa21* 3′-fragment, into the pBluescript II SK- vector to create a promoterless full-length *Myc-Xa21* gene. The 5′ end of this gene was confirmed by sequencing. This *Myc-Xa21* gene was excised with *BamH*I/*Spe*I and subcloned into the Ubi-CAMBIA-1300 vector [55] using the same enzyme sites to generate plasmid Ubi Myc-Xa21. To fuse the XA21 protein to the YFP protein, a 380-bp 3′ fragment of the *Xa21* gene was PCR-amplified using primers, 5′-TGCATCAACGCATGGAGATA-3′/5′-AATTCCATGGGAAATTCAAGGCTCCCACCTT-3′. This fragment removed the stop codon and the *EcoR*I site located in front of the stop codon. This *Xa21* 3′ PCR product was digested with *EcoR*I/*Nco*I. Meanwhile, the YFP gene was excised from the pEYFP plasmid (Clontech) using *Nco*I/*Spe*I. These two fragments were jointly cloned into pBluescript II SK-, predigested with EcoRI/SpeI, to create plasmid Xa21 3′-YFP/SK. The *Xa21* portion was confirmed by sequencing. The *Xa21* 3′-*YFP* fragment was excised with EcoRI (the second *EcoR*I site, coming from the pEYFP plasmid, is located next the end of the *YFP* gene) and used to replace the 380-bp *EcoR*I fragment in the original *Xa21* gene.

### Rice Transformation

Rice transformation was conducted as described previously [55]. Agrobacterium EHA105 was used to infect rice callus for transformation. We first generated a transgenic line expressing *Xa21* under control of its native promoter using the Phosphosmannose isomerase (*Pmi)* selectable marker. We then isolated a homozygous line XA21-4300-23A (Nat XA21) [55]. Next, we over-expressed full-length *BiP3* under the control of the Ubi promoter (BiP3 ox) using the *Hpt2* selectable marker in these XA21 homozygous lines. The transgenic Kitaake lines overexpressing BiP3 were also generated with the same construct.

### Isolation of XA21 Complex and *In Vivo* Co-Immunoprecipitation

Detached rice leaves were immersed in an *Xoo* strain PXO99Az suspension (OD600 = 0.4) for the indicated times and then harvested for protein extraction. To coimmunoprecipotate Myc-XA21 and BiP3, total proteins were extracted from 5 g of leaf tissue in 25 ml of ice-cold Extraction Buffer II [0.15 M NaCl, 0.01 M Na-phosphate pH 7.2, 2 mM EDTA, 0.1% Triton X-100, 10 mM β-mercaptoethanol, 20 mM NaF, 1 mM PMSF, 1% Protease cocktail (Sigma), 2 µg/ml leupeptin, 2 µg/ml antipain, and 2 µg/ml aprotinin]. After filtering through Miracloth (Calbiochem) followed by centrifugation twice at 13,000 g for 20 min at 4°C, the supernatant was mixed with 50 µl of agarose conjugated anti-Myc antibody (Santa Cruz) and incubated at 4°C for 2 h. The beads were then washed four times in 1 ml of Extraction Buffer II without proteinase inhibitors. The proteins were eluted with 4× Laemmli loading buffer. Protein blot analyses were performed.

### Cross-Hybridzation of BiP Overexpression and Myc-XA21 Rice Lines

The transgenic lines Ubi Myc-XA21 and Nat Myc-XA21 were used as the pollen recipients in a cross with pollen donor BiP3 ox (line 3A). Over 50 seeds were recovered from each cross. The nature of the F_1_ and F_2_ hybrid was confirmed by PCR amplification of approximately 600 bp fragment spanning part of the Myc tag and *Xa21* in the Myc-Xa21 construct using primers 5′-GAGCAAAAGCTGATTTCTGAGGAGGAT-3′/5′-ACCACCTAGCTTGTTTTCTCTGAC-3′ and approximately 500 bp fragment spanning part of the BiP3 and Nos terminator in the BiP3 ox construct using primers 5′-TGAGGAGGAGGACAAGAAGGTGAA-3′/5′-AATCATCGCAAGACCGGCAACAGG -3′.

### Immunodetection

For immunoblot analysis, proteins were separated by 8% SDS-polyacrylamide gel (SDS-PAGE). The proteins were then blotted onto a Hybond-P membrane (Amersham Pharmacia Biotech) by using SemiPhor Semi-Dry Transfer Unit (Amersham Pharmacia Biotech). For BiP detection, anti-BiP rabbit polyclonal IgG (Santa Cruz) and anti-rabbit IgG, horseradish peroxidase linked whole antibody (Amersham Pharmacia Biotech) were used as a primary and a secondary antibody at a final dilution of 1∶1,000 and 1∶5,000 for 2 h, respectively. For Myc-XA21 detection, anti-Myc mouse monoclonal IgG (Santa Cruz) and anti-mouse IgG, horseradish peroxidase linked whole antibody were used as a primary and a secondary antibody at a final dilution of 1∶1,000 and 1∶5,000 for 2 h, respectively. For actin detection, anti-actin goat polyclonal IgG (Santa Cruz) and anti-goat IgG, horseradish peroxidase linked whole antibody were used as a primary and a secondary antibody at a final dilution of 1∶1,000 and 1∶5,000 for 2 h, respectively. Bands were visualized using the SuperSignal West Pico Chemiluminescent Substrate (Pierce) according to standard protocol.

### BiP3-smGFP2 and XA21-smGFP2 Protein Constructions and Transient Expressions

PCR was performed using the BiP3-specific oligonucleotide primers, 5′-CACCATGGATCGGGTTCGCGGATGCGCG-3′/5′-GTCTAGATACAGCTCGTCATGCTCGTCGTCGAC-3′ and the Xa21-specific oligonucleotide primers 5′-CACCATGATATCACTCCCATTATTGCTC-3′/5′-GGGATCCCAGAATTCAAGGCTCCCACCTTC-3′. Using PCR, the termination codons of the *BiP3* and *Xa21* cDNA were removed. The PCR-amplified products were cloned into pENTR^TM^/D-TOPO/D vector (Invitrogen). The positive clones were verified by DNA sequencing and then using Gateway LR Clonase^TM^ (Invitrogen), moved into the coding region of soluble-modified green fluorescent protein (smGFP2) vector using Gateway LR Clonase^TM^ (Invitrogen) [31]. smGFP2-Gateway is a smGFP2 derivative with an Gateway® cassette (Invitrogen). Transient expression of green fluorescent protein (GFP) fusion constructs and H^+^-ATPase-RFP (kindly provided by Prof. Hwang) were performed by introducing the plasmid into the rice protoplasts using the PEG−mediated transformation method [31].

### Microscopy

Images were collected with an Olympus FV1000 confocal microscope. GFP was imaged under the following conditions: excitation: 488 nm; DM 405/488/543; emission: 500–530 nm. FM4-64 was imaged under the following conditions: excitation: 543 nm; DM 405/488/543; emission: 560–620 nm. Images were collected through either a 40× (numerical aperture, 1.00) or 60× (numerical aperture, 1.42) oil immersion lens. All images are the result of 2 kalman line averages and where appropriate, sequential scans were used to prevent cross talk. Images were analyzed using the Olympus Fluorview software (Ver 1.4a) and coded green (for GFP) or red (for FM4-64).

### RT-PCR

For reverse transcriptase-polymerase chain reaction (RT-PCR) analysis, total RNAs were extracted from leaves after each treatment and then the RT reaction was performed following the manual for QuantumRNA 18S Internal Standards (Ambion). PCR analyses were performed with primers pairs, 5′-GAGCAAAAGCTGATTTCTGAGGAGGAT -3′/5′-ACCACCTAGCTTGTTTTCTCTGAC-3′ (for *Myc-Xa21*), 5′-GGATCCCTGTTTGCATTTTCTGTTG-3′/5′-CATCCTTGGTTGCCTGCCTCTGC-3′ (for *BiP3*), and 5′-CCTCGCCGCGCTTCTCCTCTTCAC-3′/5′-GCCTCGGCCGTCTCCTTCATCC-3′ (for *BiP5*). The amplified products were then resolved by gel electrophoresis.

### Production of Recombinant Protein

Full-length cDNA corresponding to *BiP3* was amplified by PCR with primers 5′-CACCATGGATCGGGTTCGCGGATGCGCG-3′/5′-CTACAGCTCGTCATGCTCGTCGTCGAC-3′. PCR fragments were purified and subcloned into the pDEST15 (Invitrogen), which expresses the recombinant protein with an N-terminal GST. The resulting construct and GST expression vector were transformed into the bacterial host strain BL21 (DE3) pLysS (Invitrogen), and expression of protein was induced at midlog phase (1 mM isopropyl β-D-thiogalactosiadse, 3 h, 28°C). Recombinant proteins were purified by affinity chromatography using Glutathione Sepharose 4B (Amersham).

### *In Vitro* ATPase Activity Assay

To perform *in vitro* ATPase assays, recombinant protein GST-BiP3 or GST alone were mixed with 100 µl assay buffer (Innova Biosciences) in the presence of 5 mM MgCl_2_ and 1 mM ATP, and incubated at 25°C for 15 min [24]. Absorbance at 650 nm was measured using a plate reader (Bio-Rad) as described by the manufacturer. Activity in control reactions without ATP was subtracted from experimental reactions. Enzyme activity was calculated based on a standard curve generated from adding increasing amounts of inorganic phosphate (Pi) to the assays.

### Deglycosylation Assays

Myc-XA21 protein immunoprecipitated from Ubi Myc-XA21 line 7A-8 was used in these experiments. The deglycosylation experiments with PNGase F (Sigma) were carried out according to the manufacturer's instructions. Prior to enzymatic treatment, samples were denatured by boiling for 10 min in the presence of denaturing buffer (50 mM sodium phosphatase, pH 7.5, 0.02% SDS, 10 mM 2-mercaptoethanol). One unit of PNGase F enzyme solution was added to the reaction mixture and the reaction was incubated at 37°C for 2 h. The reaction was stopped by heating to 100°C for 5 min.

### Accession Numbers

Sequence data from this article can be found in the Rice Annotation Project Database (http://rice.plantbiology.msu.edu/) under the following accession numbers: BiP3 (Os02g02410), BiP5 (Os05g35400), Xb3 (Os05g02130), Xb10 (Os09g25070), and Xb15 (Os03g60650).

## Supporting Information

Figure S1 Rice Plants Overexpressing *Myc-Xa21* (Ubi Myc-XA21) Are Resistant to *Xoo* Strain PXO99Az. Transgenic lines carrying *Myc-Xa21* under the control of the Ubi promoter (Ubi Myc-XA21), transgenic rice carrying *Xa21* under the control of its native promoter (Nat XA21), and Kitaake wild type (Kit) were inoculated at 6 weeks of age and lesion lengths were measured 14 DAI. Each data point represents the average and standard deviation of at least four samples. Black bars in Ubi Myc-XA21 and Nat XA21 represent segregants carrying the transgene. White bars represent segregants not carrying the transgene.
(0.14 MB TIF)

Figure S2 LC-MS/MS Analysis of BiP3 and XA21. Protein bands of 140, 110, and 75 kDa were digested with trypsin and subjected to LC-MS/MS. Protein identification was performed using the TIGR database with MASCOT software [60]. (A) Over thirty peptides (red) of the 75 kDa protein matched BiP3, an ER-located member of the heat shock protein (HSP) 70 chaperone family. (B) All peptides obtained from the 140 and 110 kDa proteins are represented by black boxes below the schematic representation of the XA21 domains. SP, signal peptide; LRR, leucine rich repeats; TM, transmembrane domain; JM, juxtamembrane domain; Myc-XA21CP; cleavage product of Myc-XA21.
(0.44 MB TIF)

Figure S3 Phylogenetic Relationships among BiP Proteins from Human, Yeast, *Arabidopsis*, and Rice. (A) Phylogenetic analysis of BiPs from rice and *Arabidopsis*. Ten thousand bootstrap replicates were performed. Sequences used in this analysis were as follows: AAA52612, CAK18759, AAI12964, and AAF13605 from human; AAA34454 from yeast; AtBiP1 (At5g28640), AtBiP2 (At5g42020), and AtBiP-L (At1g09080) from *Arabidopsis*; and OsBiP2 (Os08g09770), OsBiP3 (Os02g02410), OsBiP4 (Os05g30480), OsBiP4 (Os03g50250), and OsBiP5 (Os05g35400) from rice. Both Os05g30480 and Os03g50250 are annotated as “OsBiP4” in the rice TIGR database. (B) Alignment of the peptide sequence of *Arabidopsis* BiP1 with rice BiPs. Amino acids 541 to 635 of Arabidopsis BiP1, which is used as an epitope to develop ant-BiP antibody, were aligned with rice BiPs. Gaps introduced to get the best alignment are indicated by dashes. Alignment was facilitated by the Lasergene Megalign program (DNASTAR).
(0.29 MB TIF)

Figure S4 Purified BiP3 Protein Possesses ATPase Activity. (A) The amount of inorganic phosphate (Pi), the released product of ATP hydrolysis, was plotted against the amount of GST-BiP3 (filled circles) or GST control (open circles). (B) The amount of Pi released by GST-BiP3 (0.5 Î¼M) and 30 min-boiled GST-BiP3 (0.5 Î¼M). Capped, vertical bars represent the standard deviation of values obtained from three reactions. Experiments were repeated three times with similar results. Error bars show the standard deviation of the data.
(0.08 MB TIF)

Figure S5 BiP Is Overexpressed in BiP3/Nat XA21 Double Transgenic Plants. Total protein was extracted from each plant (BiP ox/Nat XA21, Kit, and Nat XA21) and protein gel blot analysis was performed with anti-BiP and anti-actin antibodies to detect BiP3 and actin proteins, respectively.
(0.69 MB TIF)

Figure S6 Silencing *BiP3* Does Not Affect XA21-Mediated Immunity. (A) RNA accumulation of the *BiP3* transcripts in BiP3 RNAi lines (T0). Total RNA was extracted and RT-PCR was performed using BiP3-specific primers. PCR genotyping results were displayed as “+” or “−”. Control RT-PCR reactions were carried out with *18S rRNA*-specific primers. Twenty-eight PCR cycles were carried out to visualize *BiP3* and *18S rRNA*. (B) Lesion length measurements of F1 population segregating for Myc-XA21 and silenced for *BiP3* (BiP3 RNAi). The F1 segregants (*Xa21/BiP3RNAi*; +/− and +/+), BiP3 RNAi lines (−/+), and Kitaake wild type (−/−) were inoculated with *Xoo* strain PXO99Az and lesion lengths were measured 8 days post-inoculation. Nat Myc-XA21: *Xa21* driven by the native promoter. Ubi Myc-XA21: *Xa21* driven by the maize ubiquitin promoter. (C) RNA accumulation of the *BiP3* and *BiP5* transcripts in T1 segregants 3A-1 and 5A-2. Total RNA was extracted and RT-PCR was performed using *BiP3* and *BiP5*-specific primers. Control RT-PCR reactions were carried out with *18S rRNA*-specific primers. Twenty-eight PCR cycles were carried out to visualize *BiP3*, *BiP5*, and *18S rRNA*. (D) After Co-IP with increased amount of leaf tissue, protein band of 75 kDa were digested with trypsin and subjected to LC-MS/MS. Protein identification was performed using the TIGR database with MASCOT software [60]. Seven peptides (red) matched BiP5 and four of them (red and italic) were BiP5-specific peptides.
(0.44 MB TIF)

Figure S7 XA21 Is Mainly Localized to the Endoplasmic Reticulum. (A) XA21-smGFP2 and BiP3-smGFP2 fusion proteins are localized to the ER. The *Ubi Xa21-smGFP2*, *Ubi BiP3-smGFP2*, and *Ubi smGFP2* were introduced into rice protoplast cells by PEG-mediated transformation [31]. Non-transformed protoplasts were observed as a control. The expression of the introduced genes was observed 16 h after transformation. Images were collected with an Olympus FV1000 confocal microscope. The images were coded in green for smGFP2. Scale bar, 5 Î¼m. (B) The XA21-smGFP2 fusion protein is localized to the ER as well as to the presumed plasma membrane (PM). The protoplast shown at left was stained with FM4-64 (third panel, top), a marker for the PM. Images were collected with an Olympus FV1000 confocal microscope. The images were coded in green (for smGFP2) or red (for FM4-64). Scale bar, 5 Î¼m.
(2.92 MB TIF)

Figure S8 Rice Plants Carrying *Xa21-YFP* under the Control of Its Native Promoter Show Resistance to *Xoo* Strain PXO99Az. Transgenic lines carrying *Xa21-YFP* under the control of its native promoter (Nat XA21-YFP), transgenic rice carrying *Xa21* under the control of its native promoter (Nat XA21), and Kitaake wild type (Kit) were inoculated at 6 weeks of age and lesion lengths were measured 14 DAI. Each data point represents the average and standard deviation of at least four samples. Black bars in Nat XA21-YFP represent segregants carrying the transgene. White bars represent segregants not carrying the transgene.
(0.12 MB TIF)

Figure S9 Overexpressed BiP3 Does Not Affect Brassinolide-Induced Responses. (A) Seeds from XA21 and the BiP3 ox/XA21 3A-3 line were germinated on MS agar in the presence (+) or absence (−) of 0.1 ÂµM BL. Seedlings were examined 3 days after germination. (B) Effect of BL on coleoptile and root elongation in XA21 and BiP3 ox/XA21 seedlings. The plants were germinated in MS agar plates containing the indicated concentration of BL. Data presented are the means of results from four plants. Bars indicate SD.
(1.25 MB TIF)
