# Supplementary figures and images for: Overexpression of the Endoplasmic Reticulum Chaperone BiP3 Regulates XA21-Mediated Innate Immunity in Rice
Source: PLoS One. 2010 Feb 17;5(2):e9262. doi: 10.1371/journal.pone.0009262 (PMC2822859; doi:10.1371/journal.pone.0009262)

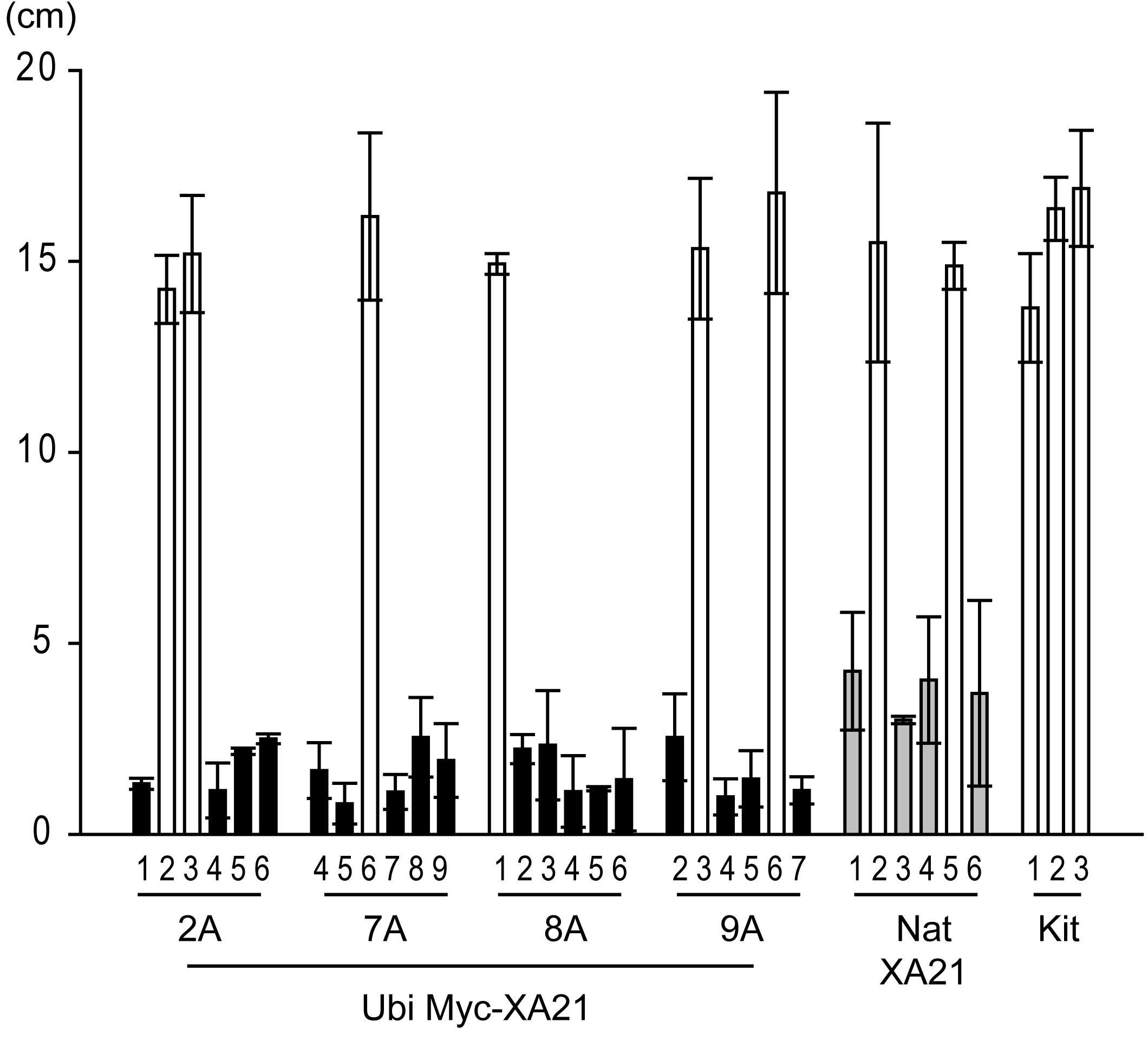

Supplement: Figure S1 — Rice Plants Overexpressing Myc-Xa21 (Ubi Myc-XA21) Are Resistant to Xoo Strain PXO99Az. Transgenic lines carrying Myc-Xa21 under the control of the Ubi promoter (Ubi Myc-XA21), transgenic rice carrying Xa21 under the control of its native promoter (Nat XA21), and Kitaake wild type (Kit) were inoculated at 6 weeks of age and lesion lengths were measured 14 DAI. Each data point represents the average and standard deviation of at least four samples. Black bars in Ubi Myc-XA21 and Nat XA21 represent segregants carrying the transgene. White bars represent segregants not carrying the transgene. (0.14 MB TIF) [file pone.0009262.s001.tif]

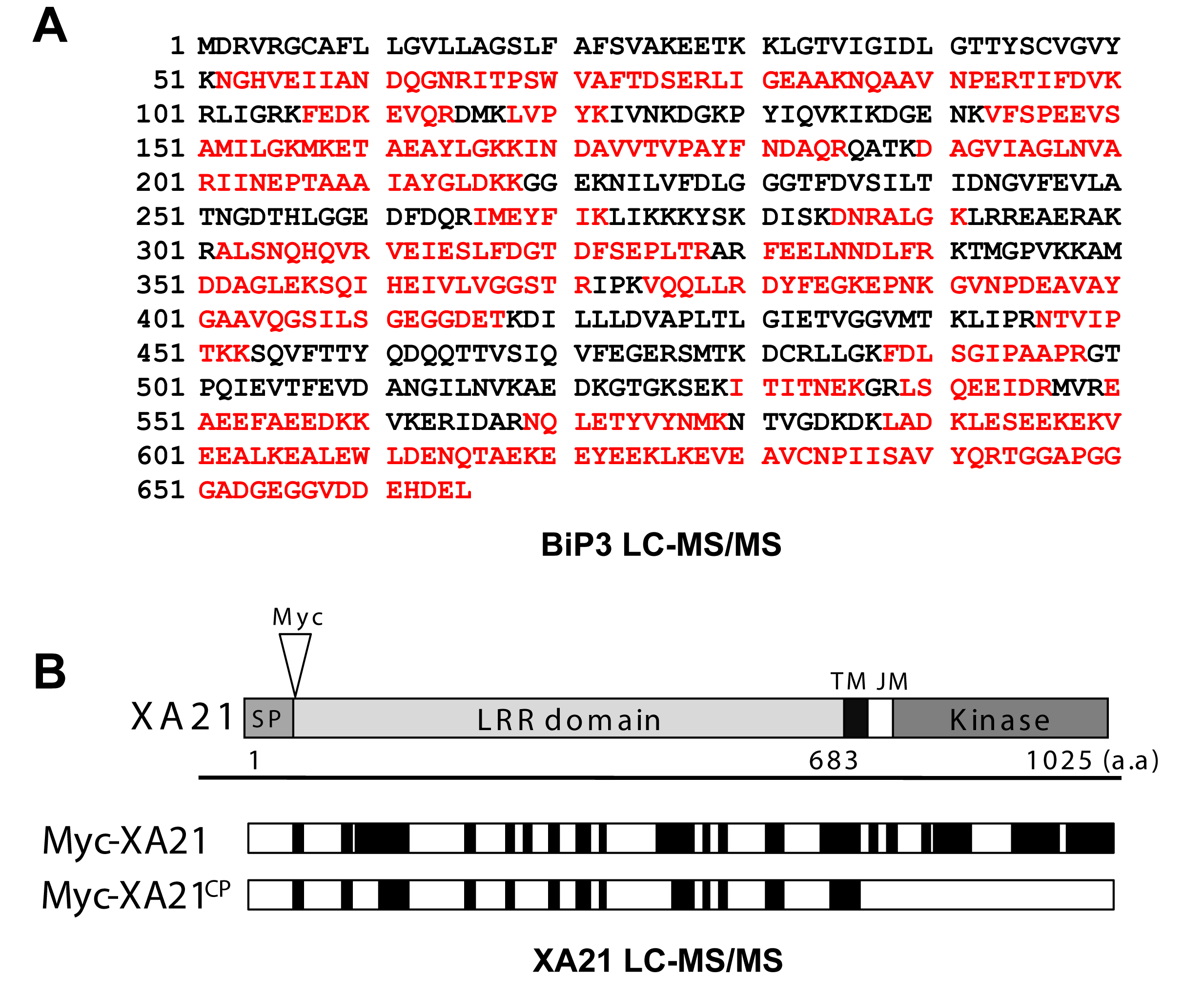

Supplement: Figure S2 — LC-MS/MS Analysis of BiP3 and XA21. Protein bands of 140, 110, and 75 kDa were digested with trypsin and subjected to LC-MS/MS. Protein identification was performed using the TIGR database with MASCOT software [60]. (A) Over thirty peptides (red) of the 75 kDa protein matched BiP3, an ER-located member of the heat shock protein (HSP) 70 chaperone family. (B) All peptides obtained from the 140 and 110 kDa proteins are represented by black boxes below the schematic representation of the XA21 domains. SP, signal peptide; LRR, leucine rich repeats; TM, transmembrane domain; JM, juxtamembrane domain; Myc-XA21CP; cleavage product of Myc-XA21. (0.44 MB TIF) [file pone.0009262.s002.tif]

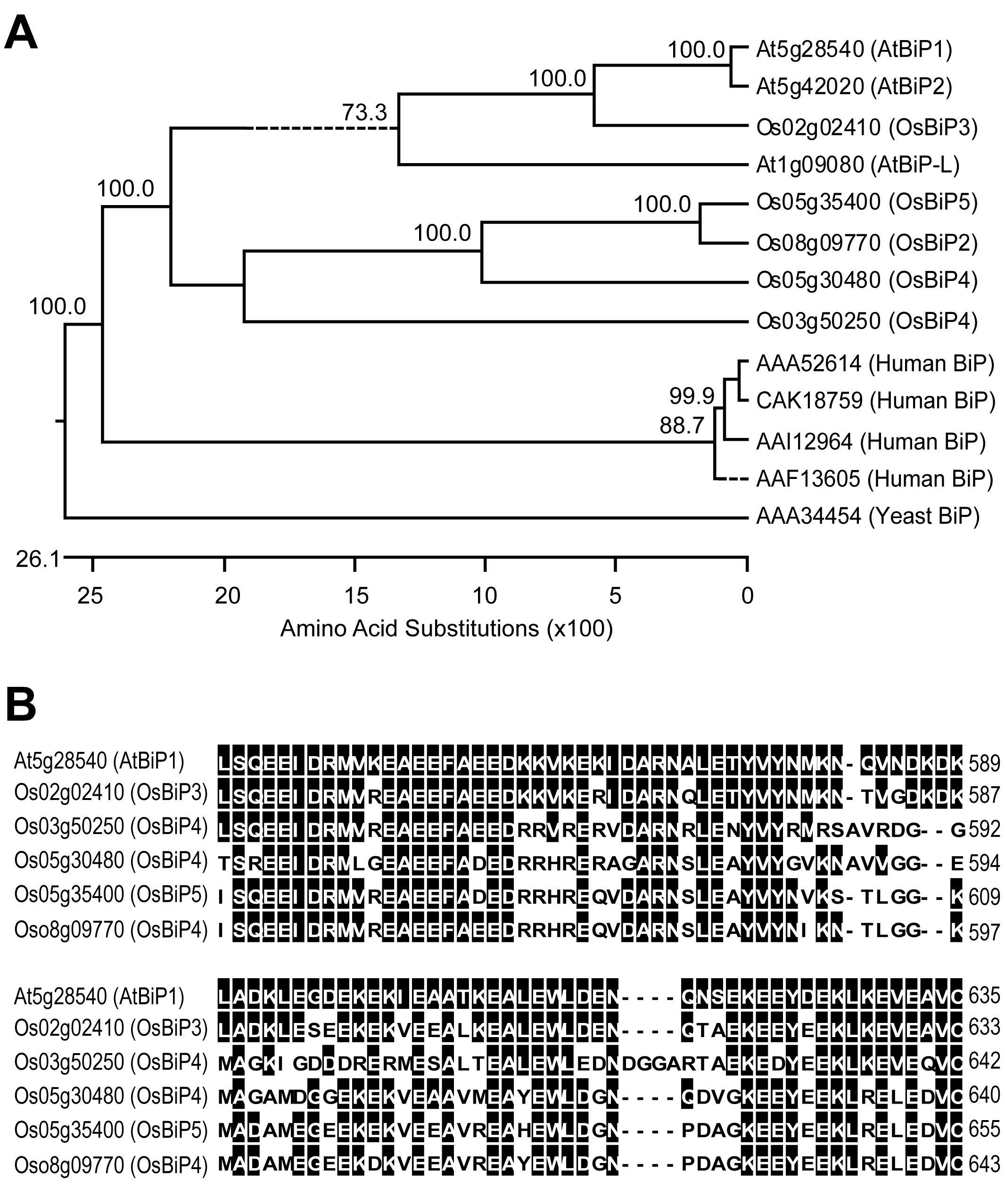

Supplement: Figure S3 — Phylogenetic Relationships among BiP Proteins from Human, Yeast, Arabidopsis, and Rice. (A) Phylogenetic analysis of BiPs from rice and Arabidopsis. Ten thousand bootstrap replicates were performed. Sequences used in this analysis were as follows: AAA52612, CAK18759, AAI12964, and AAF13605 from human; AAA34454 from yeast; AtBiP1 (At5g28640), AtBiP2 (At5g42020), and AtBiP-L (At1g09080) from Arabidopsis; and OsBiP2 (Os08g09770), OsBiP3 (Os02g02410), OsBiP4 (Os05g30480), OsBiP4 (Os03g50250), and OsBiP5 (Os05g35400) from rice. Both Os05g30480 and Os03g50250 are annotated as “OsBiP4” in the rice TIGR database. (B) Alignment of the peptide sequence of Arabidopsis BiP1 with rice BiPs. Amino acids 541 to 635 of Arabidopsis BiP1, which is used as an epitope to develop ant-BiP antibody, were aligned with rice BiPs. Gaps introduced to get the best alignment are indicated by dashes. Alignment was facilitated by the Lasergene Megalign program (DNASTAR). (0.29 MB TIF) [file pone.0009262.s003.tif]

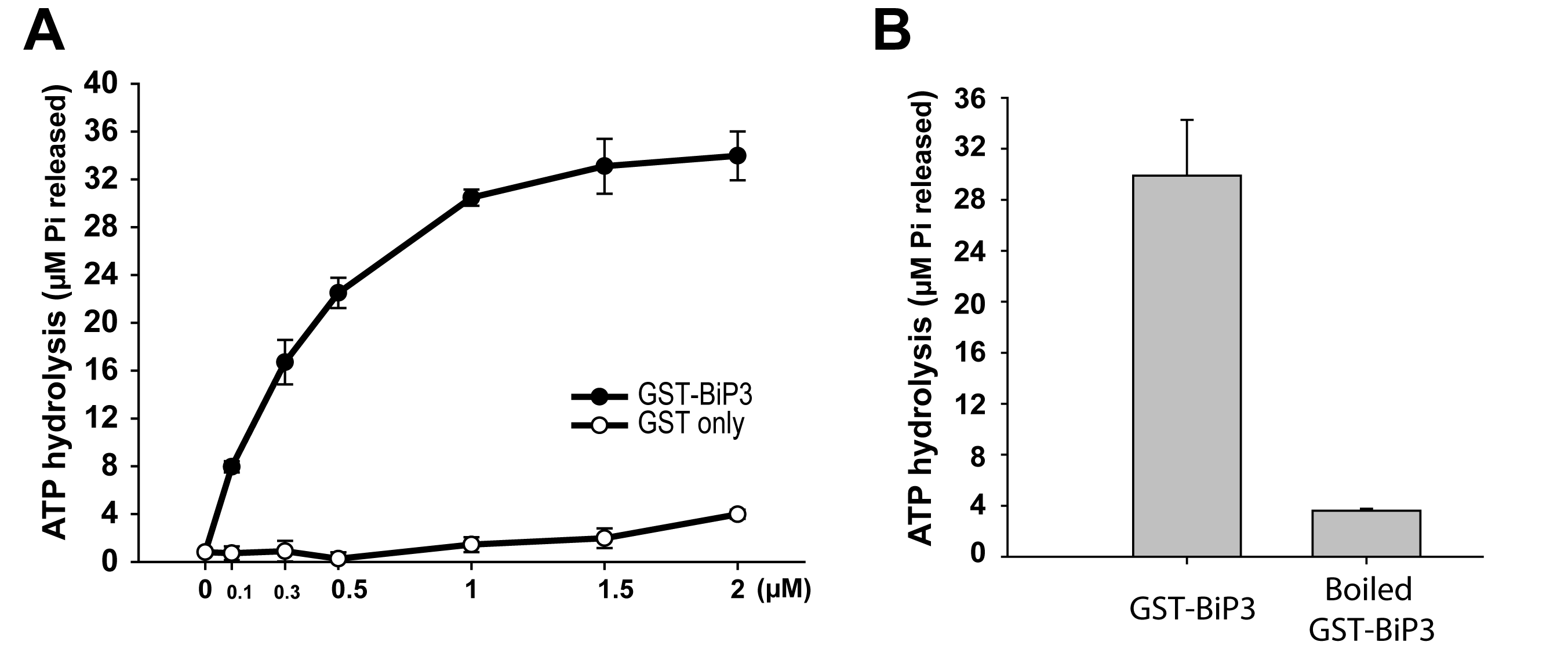

Supplement: Figure S4 — Purified BiP3 Protein Possesses ATPase Activity. (A) The amount of inorganic phosphate (Pi), the released product of ATP hydrolysis, was plotted against the amount of GST-BiP3 (filled circles) or GST control (open circles). (B) The amount of Pi released by GST-BiP3 (0.5 Î¼M) and 30 min-boiled GST-BiP3 (0.5 Î¼M). Capped, vertical bars represent the standard deviation of values obtained from three reactions. Experiments were repeated three times with similar results. Error bars show the standard deviation of the data. (0.08 MB TIF) [file pone.0009262.s004.tif]

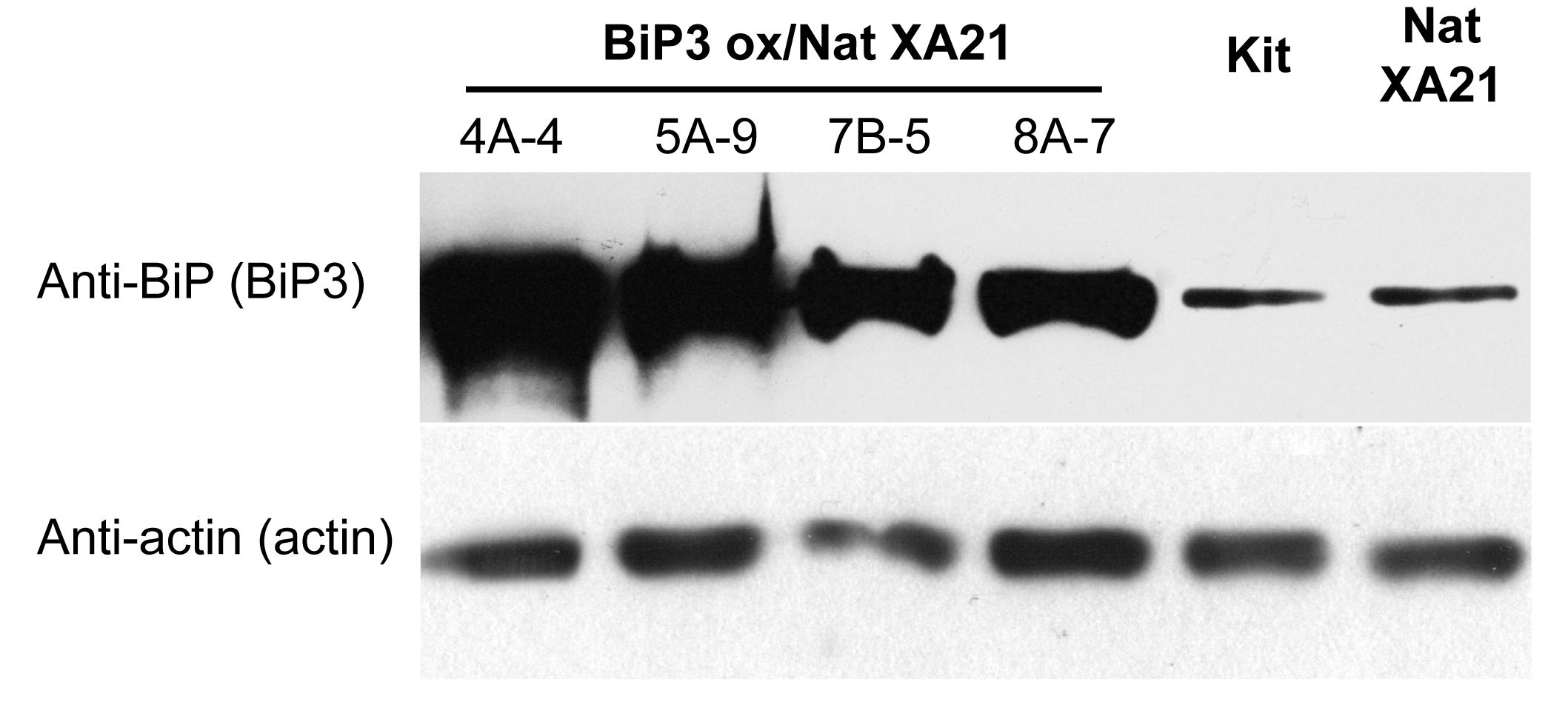

Supplement: Figure S5 — BiP Is Overexpressed in BiP3/Nat XA21 Double Transgenic Plants. Total protein was extracted from each plant (BiP ox/Nat XA21, Kit, and Nat XA21) and protein gel blot analysis was performed with anti-BiP and anti-actin antibodies to detect BiP3 and actin proteins, respectively. (0.69 MB TIF) [file pone.0009262.s005.tif]

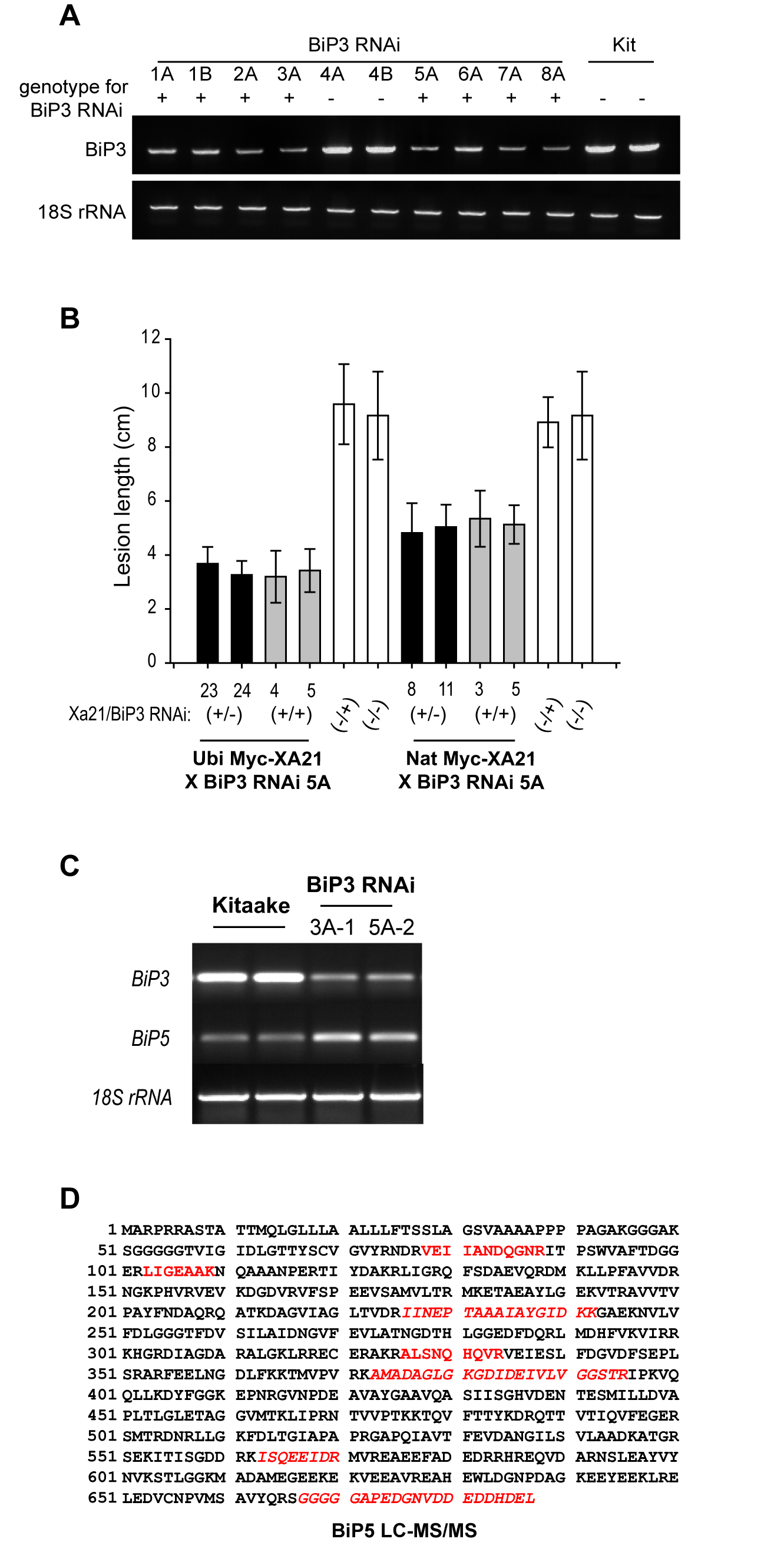

Supplement: Figure S6 — Silencing BiP3 Does Not Affect XA21-Mediated Immunity. (A) RNA accumulation of the BiP3 transcripts in BiP3 RNAi lines (T0). Total RNA was extracted and RT-PCR was performed using BiP3-specific primers. PCR genotyping results were displayed as “+” or “−”. Control RT-PCR reactions were carried out with 18S rRNA-specific primers. Twenty-eight PCR cycles were carried out to visualize BiP3 and 18S rRNA. (B) Lesion length measurements of F1 population segregating for Myc-XA21 and silenced for BiP3 (BiP3 RNAi). The F1 segregants (Xa21/BiP3RNAi; +/− and +/+), BiP3 RNAi lines (−/+), and Kitaake wild type (−/−) were inoculated with Xoo strain PXO99Az and lesion lengths were measured 8 days post-inoculation. Nat Myc-XA21: Xa21 driven by the native promoter. Ubi Myc-XA21: Xa21 driven by the maize ubiquitin promoter. (C) RNA accumulation of the BiP3 and BiP5 transcripts in T1 segregants 3A-1 and 5A-2. Total RNA was extracted and RT-PCR was performed using BiP3 and BiP5-specific primers. Control RT-PCR reactions were carried out with 18S rRNA-specific primers. Twenty-eight PCR cycles were carried out to visualize BiP3, BiP5, and 18S rRNA. (D) After Co-IP with increased amount of leaf tissue, protein band of 75 kDa were digested with trypsin and subjected to LC-MS/MS. Protein identification was performed using the TIGR database with MASCOT software [60]. Seven peptides (red) matched BiP5 and four of them (red and italic) were BiP5-specific peptides. (0.44 MB TIF) [file pone.0009262.s006.tif]

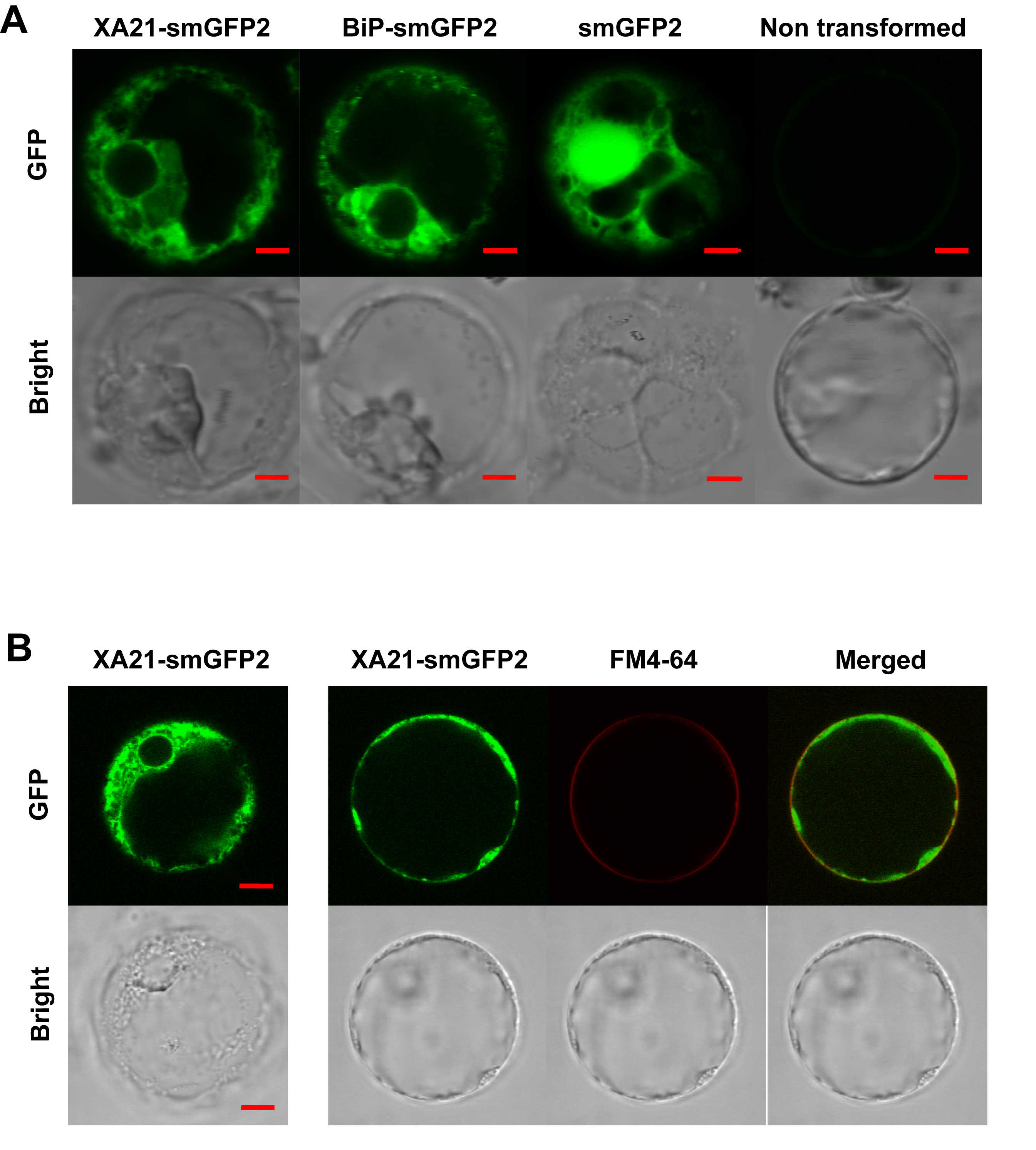

Supplement: Figure S7 — XA21 Is Mainly Localized to the Endoplasmic Reticulum. (A) XA21-smGFP2 and BiP3-smGFP2 fusion proteins are localized to the ER. The Ubi Xa21-smGFP2, Ubi BiP3-smGFP2, and Ubi smGFP2 were introduced into rice protoplast cells by PEG-mediated transformation [31]. Non-transformed protoplasts were observed as a control. The expression of the introduced genes was observed 16 h after transformation. Images were collected with an Olympus FV1000 confocal microscope. The images were coded in green for smGFP2. Scale bar, 5 Î¼m. (B) The XA21-smGFP2 fusion protein is localized to the ER as well as to the presumed plasma membrane (PM). The protoplast shown at left was stained with FM4-64 (third panel, top), a marker for the PM. Images were collected with an Olympus FV1000 confocal microscope. The images were coded in green (for smGFP2) or red (for FM4-64). Scale bar, 5 Î¼m. (2.92 MB TIF) [file pone.0009262.s007.tif]

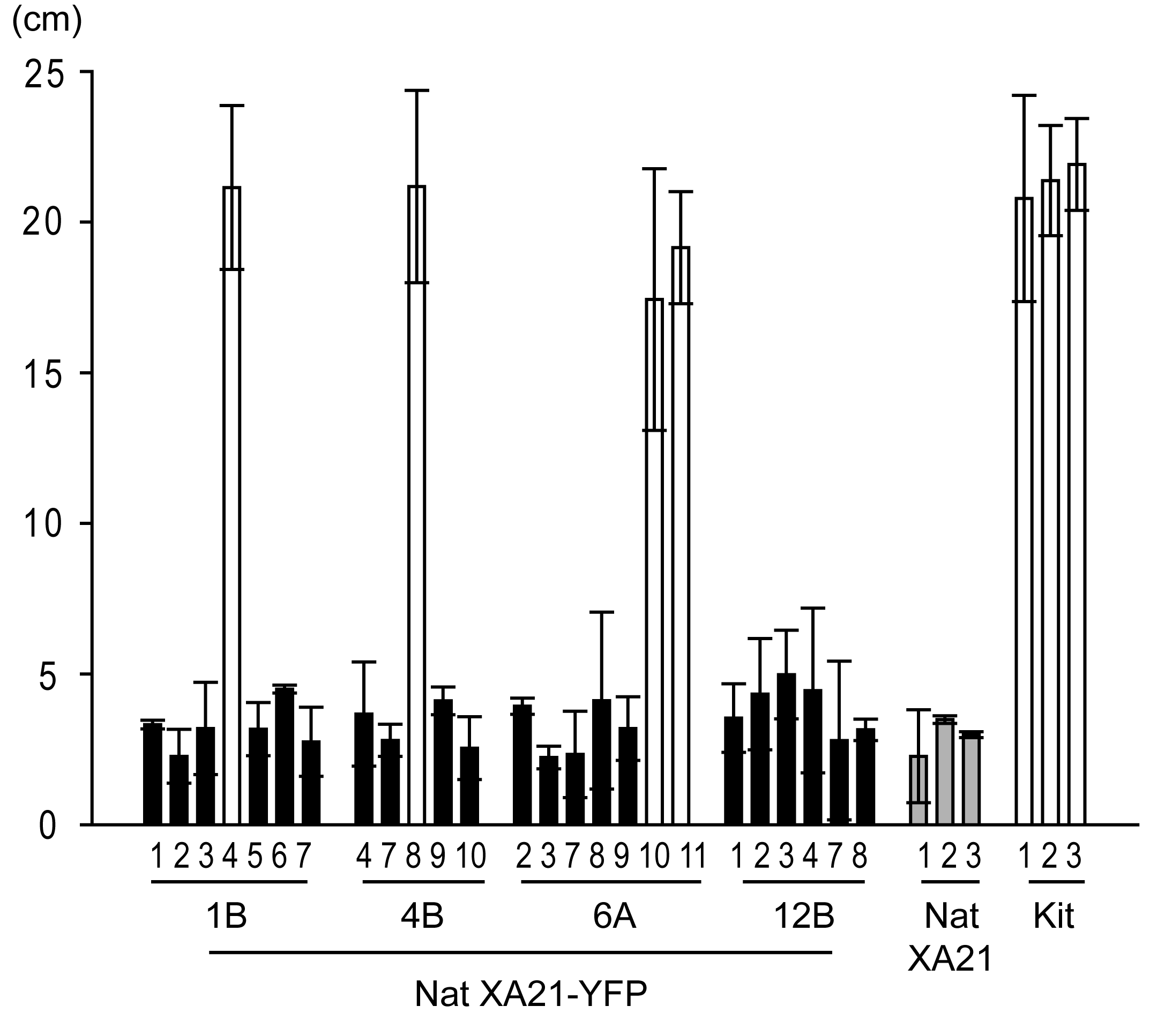

Supplement: Figure S8 — Rice Plants Carrying Xa21-YFP under the Control of Its Native Promoter Show Resistance to Xoo Strain PXO99Az. Transgenic lines carrying Xa21-YFP under the control of its native promoter (Nat XA21-YFP), transgenic rice carrying Xa21 under the control of its native promoter (Nat XA21), and Kitaake wild type (Kit) were inoculated at 6 weeks of age and lesion lengths were measured 14 DAI. Each data point represents the average and standard deviation of at least four samples. Black bars in Nat XA21-YFP represent segregants carrying the transgene. White bars represent segregants not carrying the transgene. (0.12 MB TIF) [file pone.0009262.s008.tif]

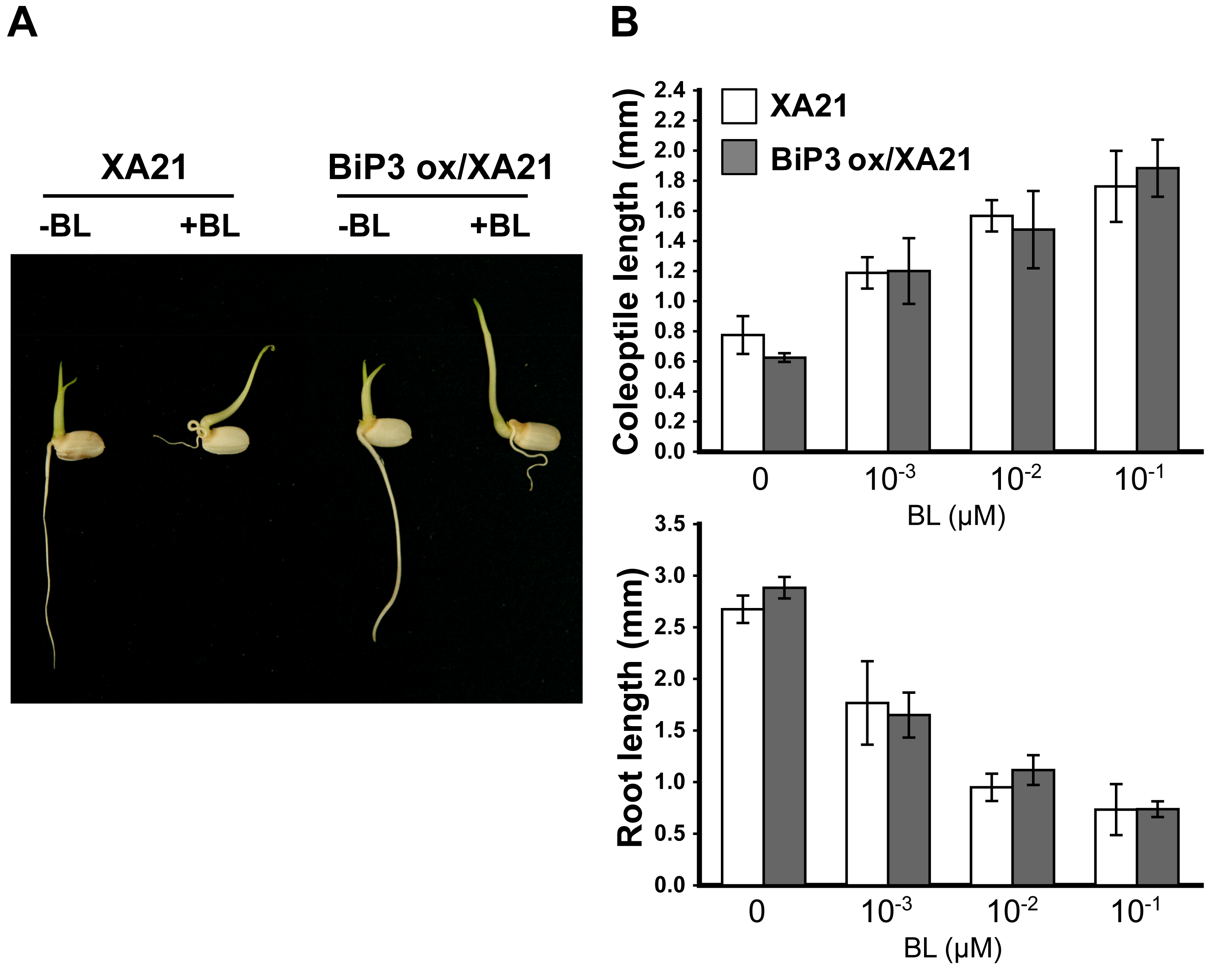

Supplement: Figure S9 — Overexpressed BiP3 Does Not Affect Brassinolide-Induced Responses. (A) Seeds from XA21 and the BiP3 ox/XA21 3A-3 line were germinated on MS agar in the presence (+) or absence (−) of 0.1 ÂµM BL. Seedlings were examined 3 days after germination. (B) Effect of BL on coleoptile and root elongation in XA21 and BiP3 ox/XA21 seedlings. The plants were germinated in MS agar plates containing the indicated concentration of BL. Data presented are the means of results from four plants. Bars indicate SD. (1.25 MB TIF) [file pone.0009262.s009.tif]
